# Lessons learned and insights from the implementation of a food and physical activity policy to prevent obesity in Mexican schools: An analysis of nationally representative survey results

**DOI:** 10.1371/journal.pone.0198585

**Published:** 2018-06-26

**Authors:** Florence L. Théodore, Jessica E. Moreno-Saracho, Anabelle Bonvecchio, María del Carmen Morales-Ruán, Lizbeth Tolentino-Mayo, Nancy López-Olmedo, Teresa Shamah-Levy, Juan A. Rivera

**Affiliations:** 1 Instituto Nacional de Salud Pública, Centro de Investigación en Nutrición y Salud, Tlalpan, Ciudad de México, México; 2 Instituto Nacional de Salud Pública, Centro de Investigación en Evaluación y Encuestas, Tlalpan, Ciudad de México, México; Public Library of Science, UNITED STATES

## Abstract

Obesity is a serious problem among children in Mexico. In 2010, the government implemented a national food and physical activity policy in elementary schools, to prevent obesity. The goal of this study is to assess the implementation of this policy, using the logic model from a descriptive survey with national representativeness at the elementary school level and based on a stratified cluster design. We used a systematic random sampling of schools (n = 122), stratified into public and private. We administered questionnaires to 116 principals, 165 members of the Food and Physical Activity Committees, 132 food school food vendors, 119 teachers, 348 parents. This study evidences a significant deviation in implementation from what had been planned. Our lessons learned are the importance to: base the design/implementation of the policy on a theoretical framework, make programs appealing to stakeholders, select concrete and measurable objective or goals, and support stakeholders during the implementation process.

## Introduction

The overweight and obesity epidemic in Mexico is a public health challenge because of its extent and widespread prevalence in all age groups [1]. National studies evidenced that schools in Mexico have turned into obesogenic environments [2] where there is an extensive availability foods unhealthy food [2, 3] and scarce opportunities for physical activity [4].

Since schools are one of the institutions where children spent most of their time, the World Health Organization [5] urged countries to transform these spaces into healthy environments and where, “healthy lifestyles” have to be promoted, which include drinking plenty of water, reducing the intake of sugar-sweetened beverages, consuming fruits and vegetables, and increasing moderate to vigorous physical activity.

In 2010, the Ministries of Education and Health launched a food and physical activity policy to prevent obesity (“policy”) aimed at promoting a “new culture of healthy living, through the development of competences for a healthy life” [6], and to create a healthy environment, that include the regulation of foods sold in schools and increase children's opportunities to be physically active during the school day. Results of a evaluation showed a lack of improvement of the nutritional content of foods and beverages [7]. Therefore, an assessment of the policy implementation became necessary to understand the reason behind those results.

Historically, evaluation policy had solely focused on biological and behavioural outcomes or knowledge intermediary indicators [8–10]; thereby giving less priority to process evaluations [11, 12]. Nowadays, in the nutrition field greater attention is paid to research oriented towards informing and improving the design and implementation of interventions [13–15]. However, little is known about the elements that contribute to the success or failure of public health policies and programs in a real-life setting [15].

There are different conceptual frameworks to analyze the implementation process of a policy. The most commonly used include the intervention Mapping Protocol [16], the RE-AIM framework [17], indicators of the implementation success, or the logic model of the Logical Framework Approach and its variants [15, 18, 19]. The logic model and its variants analyze the links between inputs, products and/or processes, and the short-term and long-term results of a program (Outcomes).

The objective of this article is to assess the implementation of an obesity prevention policy in elementary schools in Mexico and learn from it to improve the implementation process.

## Materials and methods

### Study framework: The logic model

The logic model was used to assess the two main components of the policy: healthy food and increased physical activity. Inputs correspond to resources (personnel, structures, funds) that are allocated for the operation of the policy; products/outputs are all kinds of operations and services that must be carried out in order to make the policy works. The results/outcomes are the expected changes generated. For short terms, they can be related to awareness, knowledge, attitudes, skills, intention, and motivation, etc. For intermediate terms, they are relative to behaviors or environmental changes. For or long-term or impact, there a relative to population’s health status. The goal of this NP is to tackle obesity among schoolchildren in Mexico. The present analysis uses data from a survey requested by the Ministry of Education in order to perform an outcome evaluation of the implementation of the guidelines of foods sold in schools [20] that were introduced as part of NP. The Ministry of Education also requested information about the use of six documents distributed that included information necessary to run the Program and the functioning of the two structures created to support the implementation of the program. See S1 Table. The NP, according to the logic model and based on our interpretation of available information.

### Principal inputs of the policy

The General Guidelines for Dispensing or Distribution of Foods and Beverages (‘‘Guidelines”) that establish the nutritional composition of foods and beverages authorized to be sold in elementary schools is part of this policy [21] and aimed to decrease the availability and access to energy-dense foods and beverages, to increase availability and to access to safe nutrient-dense foods (prioritizing sales of vegetables, fruits and plain drinking water) and beverages and establishing a set of instructions in terms of hygiene and safety norms.

The promotion of physical activity in schools is supported by an agreement between the Ministries of Education and Health, based on WHO recommendations that establish that children should do 30 minutes or more of physical activity per school day. The policy considers four windows of opportunity. Teachers are invited to propose games and contests during recess.

#### Information provided to schools

Six documents addressed to the school actors (principals, teachers, school food vendors, committee members, parents) were developed. Two are normative and provide information about the objectives of the policy, the Committees and their respective organization and missions. The technical documents provide advices to increase healthy food preparation and physical activity in school. The aforementioned documents are key to the dissemination of normative and technical knowledge that is necessary to drive the implementation and operation of the policy. According to the Ministry of Education, all elementary school principals around the country received at the launching, a package with a set of the six documents for them and to be distributed to the others actors. The number of sets delivered to each school is ignored. There is no information about training and/or workshops, organized by the Ministry of Education previous or posterior to the launching.

#### Organizational and human support

With the policy, two structures were created: a Food Committee (FC) and a Physical Activity Committee (PAC). Probably the most significant operational activity of both committees is the training of school food vendors or teachers to strengthen their knowledge and their ability to offer and/or promote healthy foods (FC) and physical activity (PAC). The FC is also in charge of the supervision of the implementation of the Guidelines. The activities of the PAC are related to the implementation and use of the physical activation guides for elementary school teachers and, the recovery of safe spaces at school and in surrounding areas for physical activity.

The policy states that FC and PAC should be made up of a majority of parents, as well as the principal and representatives of teachers and alumni.

### Samples of schools and actors

The data come from a descriptive survey with national representativeness at the elementary school level, based on a three-stage stratified cluster sample. We have stratified according to school sustainment (public and private). The distribution of the sample by strata was done with a proportional allocation to the number of schools per stratum. First stage uses localities as primary sampling units selected with PPS, based on the number of schools. Second stage uses a simple random selection of 4 schools per locality and a final stage simple random selection of different school actors. In order to establish representativeness, expansion factors (sampling weights) were generated at the school level by stratum and for all different school actors considered in the survey.

We obtained in June 2012, the data from 122 public and private elementary schools.

The information presented in this article comes from questionnaires administered to 116 principals, 76 members of FC, 89 members of PAC, 132 school food vendors, 119 teachers and 348 parents without any responsibility in a FC or PAC. Not all schools had committees, but all had at least, one person covering the activities related to the FC and PAC.

In Mexico, elementary schools have two shifts. We considered both. There is no major school meal program. During recess time, vendors authorized, sell the food inside the school.

### Description of the instruments used

We developed six different questionnaires for principals, Members of FC, Members of PAC, teachers, parents and school food vendors, using pre-established answer choices. Topics of the questionnaires with the stakeholders, teachers, school food vendors and parents are presented in S2. In field, we didn’t read out to the interviewees. Whenever a person provided an answer not captured by the predetermined choices, it was codified as "other". We carried out the pilot testing of the questionnaires in five schools. The pilot testing of the questionnaires was carried out in five schools.

### Statistical analysis

The proportions of responses to the different variables of interest and their 95% confidence intervals were calculated. The analyses were made adjusting by the study’s design with the module of complex samples of the SPSS v18 and Stata v12 for Windows statistical packages.

### Ethical considerations

The Ethics and Research Committees at the National Institute of Public Health (INSP) approved this study on June 6, 2012 (CI: 1999, No.1233). The Biosecurity Committee at the INSP considered the study exempt. All participants gave written consent to participate in the research project. The Declaration of Helsinki-Ethical Principles for Medical Research Involving Human Subjects was considered for this study.

## Results

### Study population

We administered questionnaires to 880 stakeholders from 122 public schools, which are representative of the 61,518 elementary schools at the national level. Table 1 describes the general characteristics of the study population.

**Table 1:** General characteristics of the study population.

	Population		Sex		School Type	
	Unweigthed, n	Weigthed, n	Men (%)	CI95%	Public (%)	95% CI
**Principals**	116	61,518	41.5	(32.3–51.4)	87.5	(86.8–88.1)
**Members of FC**	76	38,146	25.0	(14.3–40)	89.9	(88.2–91.4)
**Members of PAC**	89	46,816	45.0	(34–56.5)	89.3	(86.1–91.8)
**Teachers**	119	431,433	61.6	(48.8–73.1)	88.3	(78.4–94)
**Parents**	348	62,481	10.3	(7.4–14.3)	87.7	(87.1–88.2)
**School food vendors**	132	56,743	16.9	(11–25)	87.8	(85.5–89.7)

FC, Food Committee; PA, Physical Activity Committee.

Nearly 90% of those surveyed are from public schools. In Table 2, we summarize our main results based on the Logic Model.

**Table 2:** Primary results of the assessment of the implementation of the NP using the logic model.

Program components	Inputs	Products/outputs	Results/outcomes
**Food component**	**Technical framework**	**Availability and review of documents**	**Food availability in the school**
	The Guidelines for the regulation of food sold in schools (mandatory) are not physically present/available in every school.	Low availability of documents (in particular, the normative document) among stakeholders and especially among school food vendors, teachers and the Committee members, compared to availability among principals.	Lack of adherence to the Guidelines for the regulation of food sold in schools and ample availability of energy-dense foods, such as biscuit, little cakes, desserts and sugar-sweetened beverages, was found at the schools (Cf. Jimenez et al, 2017)
		9% of principals and 30% of Committee members were not aware of the existence of theses documents.	
		The percentage of stakeholders and food school vendors and parents that reviewed these documents is even lower.	
	**Information support**	**Committee organization and operation**	**Knowledge acquisition**
	At the launching, a package with a set of the six documents for the principal and several copies of the six documents to be distributed to teachers, members of Committees, school food venders and parents was sent to each school in the country.	Most of the Committees met between one and three times. Committees met very few times.	Acquisition by all stakeholders of a superficial technical and normative knowledge linked to (a) committee functions, (b) Guidelines for the regulation of food sold in schools.
**Objective:** To create an adequate food environment (supply and promotion) so children consume healthy foods during recess time.			
		Partial view of stakeholders about the function of the FC (heavier on supervision of fulfillment of the Guidelines for the regulation of food sold in schools).	
		Most of the committees were composed of teachers and principals. Parental involvement was low.	
	**Organizational and human support**		**Healthy lifestyle promotion**
	Food Committees (FC) and Physical Activity Committees (PAC) were supposed to be created in every school, but an important number of schools did not have them established.		Very few committees had created awareness campaigns, offered training and assistance to the school community or developed support documents to generate a culture of healthy living.
			Less than 50% of the elementary schools have a FC. In the remaining half, a teacher generally covered the FC functions.

### Product/outputs

#### Availability and review of documents

The availability of the documents among actors is low in general and varies according to the types of actor and document (Table 3).

**Table 3:** Availability and review by stakeholders, teachers, school food vendors of the six documents.

**Availability of documents according to stakeholders, teachers, school food vendors**	**Principals**	**FC Members**	**PAC members**	**Teachers**	**School food vendors**
	%	%	%	%	%
	(95% CI)	(95% CI)	(95% CI)	(95% CI)	(95% CI)
Normative documents:					
#1 Program of Action in the School Context (PASE): Guide for principals, committee members, teachers, and school food vendors.	21.1%	4.8%	10.6%	8.9%	-
	(14.3–30.1)	(1.8–12.1)	(5.6–18.9)	(4.4–17.1)	-
#2 Guidelines for the regulation of food and beverages sold in primary schools.	24.4%	10.4%	3.4%	4.2%	-
	(17.9–32.3)	(5.7–18)	(1.1–10)	(1.4–11.9)	-
Technical documents:					
#3 Guidelines to promote physical activity in the schools: Guide for principals, teachers, and committee members.	53.7%	-	59.4%	37.7%	-
	(41.4–65.6)	-	(47.8–70.1)	(27.6–49.2)	-
# 4 Guidelines for the preparation and hygiene of foods and beverages in schools: Guide for principals, teachers, food committee members, and school food vendors.	61.6%	49.9%	-	33.6%	41.8%
	(52.8–69.7)	(36.5–63.4)	-	(21.4–48.5)	(29.2–55.7)
#5 How to prepare an adequate school lunch and keep a healthy diet: Guide for parents, families, principals, and food committee members.	47.1%	41.6%	-	29.2%	-
	(39.8–54.5)	(30.7–53.4)	-	(19.5–41.2)	-
#6 Guidelines for the regulation of foods and beverages sold in primary schools: Principals, teachers, and committee members guide	28.2%	10.0%	-	11.0%	-
	(22.1–35.4)	(4.2–21.7)	-	(5.9–19.5)	-
**Reviewed documents**	**Principals**	**FC Members**	**PAC members**	**Teachers**	**School food vendors**
Normative documents:					
#1 Program of Action in the School Context (PASE): Guide for principals, committee members, teachers, and school food vendors.	20.3%	5.8%	6.9%	5.9	-
	(13.7–29)	(2.2–14.8)	(2.9–15.6)	(2.4–14)	-
#2 Guidelines for the regulation of food and beverages sold in primary schools.	23.2%	6.5%	9.2%	5.4%	-
	(15.8–32.7)	(2.9–13.7)	(4.3–18.3)	(2.2–12.6)	-
Technical documents:					
#3 Guide for physical activation. Guide for the school community (principals, teachers, members of the FC).	62.3%	10.0%	56.9%	39.7%	-
	(49.9–73.2)	(4.5–20.6)	(45.8–67.3)	(30–50.3)	-
#4 Guidelines for the preparation and hygiene of foods and beverages in schools: Guide for principals, teachers, committee members, and school food vendors.	54.3%	47.9%	-	34.0%	48.0%
	(44.7–63.6)	(34.3–61.7)	-	(23.4–46.4)	(35.8–60.5)
#5 How to prepare an adequate school lunch and keep a healthy diet: Guide for parents, families, principals, and food committee members.	48.7%	40.7%	-	24.2%	-
	(40.1–57.4)	(29.6–52.9)	-	(14.2–38)	-
#6 Guidelines for the regulation of foods and beverages sold in primary schools: Principals, teachers, and food committee members guide	33.1%	5.8%	-	6.7%	-
	(25.4–41.8)	(2.2–14.8)	-	(2.8–15.1)	-

FC, Food Committee; PAC, Physical Activity Committee.

Principals reported having a better availability to the documents, compared to other stakeholders like FC Members (21.1% Vs. 4.6% in the case of the document #1). 41.8% of school food vendors declared to have a copy of the document for preparation and hygiene of foods and beverages, but only 48% of them, had read it. Levels of review varied according to their nature and the type of actor as showed in Table 3. The principals reviewed the documents in a higher proportion, compared to members of the FC/ PAC, and the teachers. Usually, members of the FC/ PAC and teachers reviewed more the technical documents than the normative one.

#### The committees’ organization and operation

49% of the elementary schools have a FC, while in schools without one, someone from the school, generally a teacher, assumes the FC functions (Table 4). These data are unavailable for the PAC.

**Table 4:** Knowledge of the functions that must be carried out by the food and physical activity committees, according to the principals and to the members of each committee (% affirmative responses and 95% CI).

***FC*** ^a^ ***Functions***	**PRINCIPALS**		**FC MEMBERS**	
	**%**^b^	% (95% CI)	**%**	% (95% CI)
Contribute to the promotion of safe and healthy environments	21.8	(15.2–30.3)	33.8	(24–45.2)
Supervise the quality of foods and beverages sold at the school food consumption establishments (in terms of hygiene, cost, order and safety) and the fulfillment of the Guidelines for the regulation of food sold in schools	46.1	(37.1–55.4)	81.4	(70.5–88.9)
Coordinate with the school personnel to promote a new culture of health	13.1	(7.8–21)	29.0	(19.1–41.3)
Collaboration with parents/tutors, associations and the educational community, the health sector, for the development of training actions for the school community in order to promote a correct diet within and outside school.	5.1	(1.5–16.3)	14.8	(7.9–26.1)
Promote the consumption of plain drinking water	5.3	(2.4–11.3)	10.3	(4.5–21.8)
Doesn’t know	0	-	7.9	(3.5–16.8)
Other	6	(2.7–12.7)	6.9	(3.1–14.5)
***PAC***^c^***Functions***	**PRINCIPALS**		**PAC MEMBERS**	
	**%**	**CI95%**	**%**	**CI95%**
Provide incentives for the implementation and use of the physical activation guides for elementary school teachers.	36.8	(28.1–46.3)	57.1	(41.7–71.3)
Promote the recovery of safe spaces at school and in surrounding areas, to stimulate regular physical activity	29	(20.4–39.4)	39.5	(28.9–51.2)
Search for support resources, specialized personnel and basic equipment for the implementation of regular physical activity at school	11.8	(7.7–17.7)	17.8	(11.7–26.2)
Develop actions and get support from external sources (outside the school), to favor physical activation	13.4	(7.9–21.7)	15	(8.1–26.2)
Other ^d^	7.8	(3.8–15.5)	30.2	(21.4–40.9)

FC, Food Committee; PAC, Physical Activity Committee.

^a^ 49% of the elementary schools have a FC

^B^ Interview questions were open-ended. Interviewers coded responses based on pre-established categories.

^c^ % of the elementary schools with PAC are unavailable

^d^ Percentages for each category can range from 0 to 100. “Other” responses correspond to answers provided that were not related to committee functions.

### Results/outcomes

#### Knowledge acquisition

93% of principals were aware of the Guidelines and 92% believed the Guidelines were the only strategy to tackle childhood obesity in school (not shown in the table). Overall, members of the FC/ PAC, teachers and vendors showed a superficial normative and technical knowledge of the National Program (Table 4).

Indeed, principals and FC members have a partial understanding of the FC functions. They didn’t mention a large range of the functions. Usually they limited the FC functions the to the supervision of the fulfillment of Guidelines (46.1% and 81.4%, respectively). The promotion of safe/healthy environments, the consumption of plain drinking water, and the promotion of new culture of healthy living, were barely not mentioned (Table 4).

Regarding the PAC’s functions, principals have a better understanding of the committee’s promotional function. Recovering safe spaces in and around school to stimulate regular physical activity was the second PAC function that most interviewees mentioned as shown in Table 4. However, 30.2% of the functions indicated by the PAC members are not part of those established in the official documents.

Although members of the FC and principals consider that the Guidelines are a priority for this committee, their knowledge is also superficial (Table 5).

**Table 5:** Stakeholders, teachers, school food vendors and parent’ knowledge of the main content of the Guidelines for the regulation of food sold in schools.

Main aspects defined by the Guidelines	Principals		FC members		Teachers		School food vendors		Parents	
	%	CI95%	%	CI95%	%	CI95%	%	CI95%	%	CI95%
Create a healthy school lunch that meets the nutritional criteria of the Guidelines and contributes to an adequate diet for students.	77.9	(69.3–84.7)	74.2	(62.7, 83.1)	70.4	(60.3, 78.7)	64.3	(55.4–72.4)	52.7	(47.2–58.1)
The selling of vegetables and fruits should be prioritized over the selling of other kind of food	57.3	(44.5–69.2)	63.7	(51.8–74.2)	59.5	(49.3–68.8)	55.3	(46.5–63.9)	49.2	(41.8–56.6)
The selling of plain drinking water should be prioritized over the selling of juice, etc.	41.4	(33.6–49.7)	35.2	(24.7–47.3)	32.9	(21.8–46.2)	29.5	(22–38.3)	24.9	(20.1–30.5)
Beverages with non-caloric sweeteners added should only be sold at junior high schools	7.9	(4–15)	2.4	(0.5–10.9)	5.3	(2.1–12.8)	4.6	(1.9–10.4)	2.3	(1–5)
Foods should fulfill the hygiene norms for handling.	17.7	(10.2–28.9)	16.2	(9.5–26.4)	20.5	(11.9–33.1)	20.6	(13.1–30.9)	9.1	(5.9–13.8)
Only once a week the approved food (e. g. Tacos) can be substituted by a snack (hotdog, slice of pizza, etc.)	3.4	(1.3–8.5)	4.4	(1.3–13.7)	0.4	(0–3.2)	2.2	(0.6–7.6)	1.4	(0.6–3.4)
Twice a week, the prepared lunch can be substituted by liquid foods (such as yogurt)	1.7	(0.4–7)	2.1	(0.5–8.9)	1.1	(0.3–4.7)	5	(2.9–8.4)	2.1	(1–4.3)

#### Healthy lifestyle promotion

The majority of principals and members of FC and PAC reported that the activities that were mostly carried out at their schools by committees were related to the promotion of a correct diet and/or physical activity. However, according to committee members, there is, a low percentage of committees that have created awareness campaigns, offered trainings and assistance to the school community, or developed supporting documents.

## Discussion

The creation and functioning of Committees, availability and review of documents, were not implemented as designed.

The creation of both Committees did not always happen and did not play a significant role in the training processes for vendors and teachers. They failed in the dissemination of technical and normative information among the school community members. Principals/teachers/members of committee/vendors’ knowledge acquisition about healthy eating allowed in school, and the kinds of physical activity that could be done with children, was unattained. The understanding of the normative framework of this Program was also impaired. The sharing of documents with the different types of actors was done in a patchy way and even, a large percentage of Committee members were unaware of the existence of these documents. Further, when actors received any of the documents, a vast majority reported not having read them. Based on the above, all types of actors had a superficial knowledge and understanding of the policy that, created barriers in the flow of implementation.

The lack of concrete objectives, goals and clearly stated roles that hinder the translation of the policy activities to concrete and simple actions, was on other barrier for the implementation of the policy. Most FC chose to focus on the Guidelines, leaving to a second place the promotion and training activities. In addition, the members of the FC, almost did not mention the promotion of water consumption among children as one of the FC’s main goals, despite the fact that the reduction of the consumption of sugar-sweetened beverages (SSB) is a national priority.

Despite the intention to involve a wide range of schools actors through committees as a strategy for the diffusion of information and create commitment about healthy lifestyles to be adopted, in practice, a low involvement of families, school food vendors, and children was documented in the operation and organization of the Committees.

All above failures displayed had hampered the attainment of the expected outcomes for building a culture of healthy living and the creation of a healthy environment.

### Lessons learned

Based the Mexican experience and others from all over the word [22–27], we identified the following lessons:

- **Theoretical framework and its importance**

A framework is essential to picture the possible pathways leading to change, understand how the program will lead to results, and clearly articulate how the program activities will lead to immediate outputs and outcomes/results (clearly established process and outcome indicators), as well as track progress and make course corrections and solve problem that might arise during implementation.

- **Appealing programs**

The difficulty encountered by both Committees in involving parents reflects a widely reported issue in the international scientific literature [28–30] related to parents’ lack of time to participate in meetings and/or lack of resources to buy and prepare healthy foods [31] and lack of awareness of the importance of eating healthily and physical activity behaviours [5]. The policy did not adopt an attractive strategy for parents to keep them engaged and failed in convince them about its importance to children and documentation was not enough.

- **Measurable objectives**

The confused nature of this policy starts from the formulation of its general goal: “To promote a new culture of healthy living through the development of skills for a healthy life”. This formulation is abstract and difficult to measure. The general design of this policy is complex because of a large number of actors involved and many dimensions/goals considered.

It is noteworthy that principals/members of FC and PAC/teachers equate the Program with the Guidelines whereas its scope is much broader. Instead of establishing very broad objectives such as the promotion of healthy diets––thus leaving much room for imagination and alternative interpretations––the policy/program should formulate a few concrete, simple, and measurable objectives. Horodyska et al. [26], based on their systematic review, also identified “simplicity as a factor facilitating implementation”.

- **Support stakeholders**

The policy is structured in such a way that most of the operational responsibility falls upon the individual schools actors. It hinges on some big assumptions expecting that the delivery of materials will be enough to guarantee adequate implementation that translates in expected outcomes.

The policy makes no provisions to train and support schools actors in the implementation process. Supporting stakeholders and overseeing programs’ implementation is a key to success, as evidenced in a strategy for adolescent obesity prevention in the Netherlands [22]. The strategy outlines, step by step, how teachers can get involved in the program [27]. In a systematic review of scientific articles and stakeholder documents [26], authors established a list of 83 conditions for successful implementation of interventions and policies aimed at the prevention of obesity. Training for local implementers in its different forms (formal trainings, workshops, certifications, etc.) are facilitators for the adoption by the implementers of the policy.

#### Limitations of the study

Several study limitations can be acknowledged. First, it was not possible to confirm the program’s theoretical grounding. We had to choose a theoretical framework (Cf. S1 Table) and organize the data accordingly to understand the implementation of the NP and be able to detect failures and successes. Second, the principal aim of the survey was to inform Ministries of Education on the evaluation of the implementation of the Guidelines [20] and the use of the materials that are part of the NP. In a context of limited resources (financial, human), we added a few questions in the original survey about the implementation of the NP and the functioning of the committees, which was for us an extraordinary opportunity to generate information with national representativeness. However, we were not able to do it with the same depth as would have been necessary to assess the implementation. Third, it would have been illuminating to complement this data with a qualitative component including school actors involved in the implementation, to explore their experience. And fourth, in a context in which stakeholders felt they were being evaluated and had to demonstrate that they implemented the Program, it was extremely difficult to collect information that could reflect, in a precise manner, the schools’ real experiences.

## Conclusion

Although the Mexican government tried to tackle obesity in the school system, this study evidenced a number of difficulties that have seriously compromised the expected outcome. Major changes should be made in order to strengthen the policy.

## Supporting information

S1 Table The Program according to the logic model. (DOCX)

S2 Table Summary information and tools obtained from the study. (DOCX)

S3 Table Level measurement and indicators used in the evaluation. (DOCX)

S1 Database Of the project. (ZIP)

S1 Survey Questionnaires Of the project. (DOCX)
