# Supplementary material for: Lessons learned and insights from the implementation of a food and physical activity policy to prevent obesity in Mexican schools: An analysis of nationally representative survey results
Source: PLoS One. 2018 Jun 26;13(6):e0198585. doi: 10.1371/journal.pone.0198585 (PMC6019747; doi:10.1371/journal.pone.0198585)
Supplement: S1 Table — (DOCX) [file pone.0198585.s001.docx]

**S1 Table.** The Mexican obesity prevention Program for children according to the logic model

| **Program Components** |  | **Inputs** |  | **Products/outputs** |  | **Results /outcome** |
| --- | --- | --- | --- | --- | --- | --- |
|  |  |  |  |  |  |  |
| **Food component objective:**  Create conditions (supply and promotion) so that during recess, children consume healthy foods. |  | ***Technical framework***  - Introduction of the regulation of food and beverages to be sold at schools, following nutritional regulation criteria known as Nutritional Guidelines (mandatory).  - Introduction of the Agreement of Children having to do in school a minimum of 30 minutes of physical activity per day.  ***Information support***  - Design and delivery in schools of normative and technical documents on food and physical activity (total= 6)  - Ministry of Education sends once, to each school of the country, a package with several copies of the six manuals for their distribution among school implementors/stakeholders.  - Principals are responsible for distributing these manuals in their school.  - No reference to, or records of, a national training  ***Organizational and human support***  - Creation of two structures with specific characteristics (composition, operating mode) within the framework of the Policy: the Food Committee (FC) and the Physical Activity Committee (PAC). Committees are composed by a diversity of school community actors (teachers, director, parents, alumni). They have to meet from one up to three times a year.  - Three main types of activities of the committees are established; (a) the operation and organization of the committees, (b) promotion of healthy lifestyles in the school community (c) the construction of a healthy school environment. |  | ***Availability and the review of the documents***    Principals, FC and PAC’s members, teachers, school food vendors and parents have and reviewed the Program documents.  ***Committee organization and operation***  - Creation in elementary schools of the FC and PAC  - FC meets between one and three times a year.  - Committees are composed of a diversity of stakeholders (teachers, principals, parents, alumni)  - Committees promote healthy lifestyles in the school community and implement activities that contribute in the construction of a healthy school environment. |  | Creation of a **new health culture through:** School Healthy environments  - Large availability of Healthy food in school. - Children perform a minimum of half an hour of physical activity in school per day. |
|  |  |  |  |  |  | Development of skills for a healthy life (ex: Knowledge, skills and values) of the members of the school community (teachers, children, director, parents, etc.).  - Stakeholders **know** the policy in detail, operation and mission of Committee and technical aspects (Nutritional Guidelines, physical activity).  - Creation of awareness campaigns, offered training and assistance to the school community or developed support materials in order to generate a health culture.  Adoption by the members of the school community of a Healthy lifestyle (healthy food and regular physical activity.  Organization of championship, o any kind of manifestation in order to promote physical activity.  **Control and decrease in the prevalence of childhood obesity** |
| **Physical activity component**  **Objective:**  Create conditions (supply and promotion) for children to do regular physical activity during the school day. |  |  |  |  |  |  |
