# Supplementary material for: Lessons learned and insights from the implementation of a food and physical activity policy to prevent obesity in Mexican schools: An analysis of nationally representative survey results
Source: PLoS One. 2018 Jun 26;13(6):e0198585. doi: 10.1371/journal.pone.0198585 (PMC6019747; doi:10.1371/journal.pone.0198585)
Supplement: S2 Table — (DOCX) [file pone.0198585.s002.docx]

**S2 Table. Summary information and tools obtained from the study**

| **Actor** | **Instrument components** |
| --- | --- |
| Principal | - I.D. record/file with school’s general data - Information about the reasons that generated the General Guidelines for the dispensing or distribution of foods and beverages - Level of information for the application of the nutritional criteria - Information about the use of the educational materials - Information about the operation and activities of the FC - Conditions which facilitate or stand in the way of the application of the general guidelines for the dispensing or distribution of foods and beverages in school food stores - Information about PAC operation and activities - Conditions that facilitate or stand in the way of the application of the guidelines when promoting the regular practice of physical activity - Health promotion for the school community |
| Committee members | - Information related to the application of the general guidelines for the dispensing or distribution of foods and beverages - Level of information for the application of the nutritional criteria - Information about the use of the materials - Information about PAC operation and activities - Identifying support materials for the implementation of the guidelines (diet and physical activation) - Use of support materials - Level of information to apply the nutritional criteria (same format as the one applied to the principal) - Actions carried out by the FC - Document actions carried out by the PAC - Knowledge and appropriation of the FC Manual |
| Food vendors | - Level of information to apply the nutritional criteria - Food preparation and hygiene (practices) - Process to evaluate compliance with the guidelines and changes that were made in order to achieve application of the Guidelines. |
|  |  |
|  |  |
| Parents | - Level of information on the Guidelines - Availability of support material for the implementation of the guidelines |
| Children | - Document lunch (foods and beverages) content - Level of information on the guidelines - Dietary behavior at school (and whether healthy dietary practices are being adopted) - Physical Education (Number of classes per week and frequency) |
| Observation | - Products being sold at schools - Document the preparation, management and preservation of foods and beverages / Document sanitation and safety of the school food establishments. - Access and availability of plain drinking water. - Structured observation of physical activation |
| Cross-sectional analyses of all questionnaires | - Conditions that stand in the way or facilitate the application of the general guidelines for the dispensing or distribution of foods and beverages at the school food consumption establishments and promotion of physical activation. |
