# Supplementary material for: Lessons learned and insights from the implementation of a food and physical activity policy to prevent obesity in Mexican schools: An analysis of nationally representative survey results
Source: PLoS One. 2018 Jun 26;13(6):e0198585. doi: 10.1371/journal.pone.0198585 (PMC6019747; doi:10.1371/journal.pone.0198585)
Supplement: S3 Table — (DOCX) [file pone.0198585.s003.docx]

**S3 Table. Level measurement and indicators used in the evaluation**

|  | **Aspect to be evaluated** | **Level measuring** | **Tool** | **Indicator** |
| --- | --- | --- | --- | --- |
| Physical activation | Progress in implementing activation session | School | Observation of activation session | - Average duration of observed Physical Activation sessions.  - Percentage of schools with a daily physical activation practices observed (more than or equal to 30 minutes). |
| Food | Access and availability of drinking water | School | Observation | -Percentage of schools with running water fountains  - Numbers of drinking fountains with running water and expressed by a number of children (e.g. Number of drinking fountains per 10 children)  - Percentage of schools with carboy of water into the classroom  - Number of carboy with water in classroom between total of classrooms  - Percentage of schools with carboy in common areas    Percentage of schools that sell bottled water.  - Number of bottles of water that are offered expressed by number of children |
|  | Offer food and beverage | School | Observation  in recess | Percentage of total food and drinks available in school that meet the criteria of the guidelines according to the categories established therein:  -Food prepared  -Milk  -Yogurt And fermented dairy foods  Saps fruit and vegetable juices  -Nectars  -Food Liquid soy  -Snack  -Biscuit, Pastries, sweets and desserts  -Drinks For preschool and primary |
|  | Preparation, management and conservation of food | School | Observation | Percentage of total school (shops or cooperatives) that meet the criteria |
|  | Committees | School | Questionnaire for directors | - Percentage of directors who know the reason for the implementation of the guidelines  - Percentage of directors who know the Food guidelines  - Percentage of directors who have educational materials of the FC and PAC.  - Percentage of directors showing copy of the list of foods that meet nutritional criteria  - Percentage of schools with an FC  - Percentage of schools with a PAC |
|  | Level information for the application of nutritional criteria | Directores  Profesores  Profesores/  Padres integrantes de CEPS/Comité de consumo  Padres  Expendedor/ vendedor  Niños | Questionnaire for  Directors/teachers/ members of PAC or FC/ vendors, children. | - Percentage of directors that identify the 80% criteria  Nutrimental the guidelines  - Percentage of teachers / parents FC members identifying all nutrient criteria guidelines  - Percentage of parents who know the components of a healthy lunch  - Percentage of vendors who have received a course, training or discussion regarding the guidelines.  - Percentage of parents who have received a course, training or discussion regarding the guidelines. |
|  | P.E. Class | Group | Questionnaire for teachers not a members of PAC | Percentage of groups with physical education teacher  Percentage of groups that meet the recommended number of classes per week as recommended in primary and secondary. |

* The values obtained will be multiplied by a factor (eg. 10, 100) for better understanding
