# Supplementary material for: Lessons learned and insights from the implementation of a food and physical activity policy to prevent obesity in Mexican schools: An analysis of nationally representative survey results
Source: PLoS One. 2018 Jun 26;13(6):e0198585. doi: 10.1371/journal.pone.0198585 (PMC6019747; doi:10.1371/journal.pone.0198585)
Supplement: S1 Survey Questionnaires — (DOCX) [file pone.0198585.s005.docx]

**INSTITUTO NACIONAL DE SALUD PÚBLICA**

**CENTRO DE INVESTIGACIÓN EN NUTRICIÓN Y SALUD**

1. **Questionnaire with FC Members**

**ESTUDIO SOBRE LA APLICACIÓN DE LA ETAPA II DE LOS LINEAMIENTOS GENERALES PARA EL EXPENDIO O DISTRIBUCIÓN DE ALIMENTOS Y BEBIDAS EN LOS ESTABLECIMIENTOS DE CONSUMO ESCOLAR DE LOS PLANTELES DE EDUCACIÓN BÁSICA**

**CUESTIONARIO PARA INTEGRANTES DEL COMITÉ DE ESTABLECIMIENTOS DE CONSUMO ESCOLAR**

**FICHA DE IDENTIFICACIÓN**

1. Código del entrevistador |__|__| 2. Código de la escuela |__|__|

3. Clave de la escuela |__|__| 4. Localidad |__|__|

5. Municipio |__|__| 6. Estado |__|__|

7. Nombre de la escuela: __________________________________________________________

8. Turno: ______________________

9. Dirección de la escuela: ________________________________________________________________________________________________________________________________________________________________

10. Código del formato: |__|__|__|

11. Código del encuestado: |__|__|__|__|__|__|

12. Fecha de aplicación del cuestionario |__|__|__|__|__|__|

Día Mes Año

1. Usted es integrante o cubre las funciones de :

CEPS |___| (1. Si, 2.No, NA cuando no hay CEPS)

Comité de Establecimientos de Consumo Escolar |___| (1. Si, 2.No, NA cuando no hay Comité de Establecimiento)

Comité de Impulso de la Activación física |___| (1. Si, 2.No, NA cuando no hay Comité de Impulso)

1. ¿Desde cuándo? (**Poner duración en meses o NA**)

CEPS |___|

Comité de Establecimientos |___|

Comité de Impulso |___|

1. Usted está involucrado en calidad de :

Profesor de grupo |___| (1. Si, 2.No, o NA)

Profesor de educación física |___| (1. Si, 2.No, o NA)

Madre/padre de familia o tutor |___| (1. Si, 2.No, o NA)

Otro (**especificar**) _______________

1. Pertenencia a la asociación de padres de familia de la escuela **|___|(solamente para madre/padre de familia)**
2. Nivel de escolaridad |___| (para todos)
3. Sexo |___| (1. Mujer, 2. Hombre)
4. Edad |___|___| (años)

**INFORMACIÓN RELATIVA A LA APLICACIÓN DE LA ETAPA II DE LOS LINEAMIENTOS GENERALES PARA EL EXPENDIO O DISTRIBUCIÓN DE ALIMENTOS Y BEBIDAS EN LOS ESTABLECIMIENTOS DE CONSUMO ESCOLAR DE LOS PLANTELES DE EDUCACIÓN BÁSICA**

1. ¿Conoce Usted los Lineamientos generales para el expendio o distribución de alimentos y bebidas en los establecimientos de consumo escolar de los planteles de educación básica? **(No leer las opciones, esperar que responda y marcar la respuesta)**
   1. Sí
   2. No

**Leer la siguiente oración de explicación:**

Los lineamientos son un conjunto de acciones de la SEP sobre la venta de alimentos y la realización de actividad física en la escuela.

1. ¿Conoce usted qué propició la creación de estos Lineamientos generales para el expendio o distribución de alimentos y bebidas? **(No leer las opciones, esperar que responda y marcar la o las opciones que se asemejen a su respuesta)**
2. Sobrepeso/obesidad
3. Diabetes
4. Sedentarismo
5. Falta de disponibilidad de agua simple potable
6. Amplia disponibilidad de alimentos con alto contenido calórico
7. No sabe
8. Otro (especificar)_____________________________________________________
9. ¿En qué consiste la aplicación de los Lineamientos generales para el expendio o distribución de alimentos y bebidas? **(No leer las opciones, esperar que responda y marcar la o las opciones que se asemejen a su respuesta)**
10. Regular la venta de alimentos y bebidas dentro de la escuela (o cualquier opción relacionada solo con alimentación y nutrición).
11. Mejorar la práctica de actividad física en las escuelas (o cualquier opción relacionada sólo con activación o actividad física).
12. Promover un refrigerio saludable proporcionando información a los padres.

Privilegiar la disponibilidad y consumo de agua simple potable

1. No sabe
2. Otro (especificar)_____________________________________________________
3. ¿La comunidad educativa ha recabado recursos económicos para la aplicación de los lineamientos generales para el expendio o distribución de alimentos y bebidas? **No leer las opciones, esperar que responda y marcar la respuesta)**
   1. Sí
   2. No
   3. No sabe
4. ¿La aplicación de los **lineamientos** generales para el expendio o distribución de alimentos y bebidas implicó algún cambio para su escuela? **(No leer las opciones, esperar que responda y marcar la respuesta)**
   1. Sí
   2. No **(pase a la pregunta 7)**
   3. No sabe **(pase a la pregunta 7)**
5. ¿Cuáles fueron los cambios? **(No leer las opciones, esperar que responda y marcar la o las opciones que se asemejen a su respuesta)**
6. Cambios relacionados con la venta de alimentos
7. Cambios relacionados con la venta de bebidas
8. Cambios relacionados con la disponibilidad de agua simple potable
9. Cambios relacionados con la activación física regular
10. Cambios relacionados con la promoción de una alimentación correcta
11. Cambios relacionados con la promoción de activación física regular
12. Cambios relacionados con el incremento de la participación del comité
13. Otro (especificar) _____________________________
14. ¿Podría mencionar en qué etapa de aplicación de los lineamientos generales para el expendio o distribución de alimentos y bebidas nos encontramos en este ciclo escolar? (**No leer las opciones, esperar que responda y marcar la respuesta**)
15. Etapa I
16. Etapa II
17. Etapa III
18. No sabe
19. Otro (especificar)
20. ¿Conoce Usted los cambios que diferencian la etapa I de la II de los lineamientos generales para el expendio o distribución de alimentos y bebidas? (**No leer las opciones, esperar que responda y marcar la respuesta**)
21. Sí
22. No **(pase a la pregunta 10)**
23. ¿Cuáles son? **(No leer las opciones, esperar que responda y marcar la o las opciones que se asemejen a su respuesta)**
    1. Disminución de **grasas, sales y azúcares** en los productos que se expenden en los establecimientos de consumo escolar
    2. Se **prioriza** la venta de **alimentos preparados**
    3. **Disminución de porciones** en los alimentos que se expenden
    4. **Eliminación** de la venta de algunos productos **industrializados**
    5. **Aumento** de consumo de **verduras y frutas**
    6. Otro. (Especificar)________________________________
24. Dentro de la escuela ¿Alguna persona o personas supervisan la aplicación de los lineamientos generales para el expendio o distribución de alimentos y bebidas? (**No leer las opciones, esperar que responda y marcar la respuesta**)
    1. Sí
    2. No (**pase a la pregunta 12)**
    3. No sabe (**pase a la pregunta 12)**
25. ¿Quién supervisa el cumplimiento? (**No leer las opciones, esperar que responda y marcar la o las opciones que se asemejen a su respuesta)**
26. Director
27. Profesores
28. Asociación de padres de familia
29. Supervisor de la zona escolar
30. Integrante(s) del Comité de Establecimiento al Consumo Escolar
31. Otro (especificar):
32. ¿Cuenta usted con un listado de alimentos y bebidas que cumplen con los criterios nutrimentales de los Lineamientos? (**No leer las opciones, esperar que responda y marcar la respuesta**)
33. Sí
34. No **(pase a la pregunta 15)**
35. En caso de SI, comprobar y especificar:
36. La mostró impresa
37. La mostró electrónica
38. Accede por internet (preguntar el link)
39. Otro (especificar) ____________________

Pase a la pregunta 15

1. En caso de NO comprobar que cuenta con el listado, explorar la razón:
2. No sabe a qué listado se refieren
3. No lo tienen
4. Lo conocen pero no lo usan
5. Otro (especificar) _________________________________________
6. ¿Me podría mencionar las estrategias que se aplican en la escuela para facilitar una alimentación correcta? (**No leer las opciones, esperar que responda y marcar la o las opciones que se asemejen a su respuesta)**
   1. Difusión y promoción de la alimentación correcta en el aula, en la escuela y en el hogar
   2. Sensibilización, capacitación y asesoría
   3. Diseño de materiales de apoyo (carteles, trípticos, folletos, películas, etc.)
   4. Diseño e implementación del programa de trabajo
   5. Supervisión y seguimiento a la aplicación de las estrategias y acciones
   6. Otras (especificar)

**NIVEL DE INFORMACION PARA LA APLICACIÓN DE LOS CRITERIOS NUTRIMENTALES**

1. ¿ Podría mencionar cuáles son los principales criterios nutrimentales que definen los Lineamientos generales para el expendio o distribución de alimentos y bebidas? **(No leer las opciones, esperar que responda y marcar la o las opciones que se asemejen a su respuesta)**

| Criterio | Sí Menciona |
| --- | --- |
| Conformación de un refrigerio escolar que contribuya a una alimentación correcta para los alumnos. |  |
| Priorizar la venta de verduras y frutas. |  |
| Priorizar la venta de agua simple potable. |  |
| Priorizar la venta de alimentos preparados que favorecen una dieta correcta. |  |
| Que las características nutrimentales de los alimentos y bebidas sean de acuerdo a la cantidad de energía y nutrimentos que requieren los estudiantes para promover y mantener su salud. |  |
| Que las bebidas con edulcorantes no calóricos se vendan solo en secundarias *(en presentaciones de 250 ml y sin cafeína).* |  |
| Que los alimentos cumplan con las normas higiénicas de preparación y consumo. |  |
| Que 1 vez por semana, se podrá sustituir el alimento preparado del refrigerio por alguna botana, pastelillo o confite |  |
| Que 2 veces por semana, se podrán sustituir las preparaciones del refrigerio por alimentos líquidos *(leche semidescremada, descremada, yogurt, alimentos líquidos de soya, jugos o néctares)*. |  |

1. ¿Podría mencionar qué alimentos debe incluir un refrigerio escolar saludable? **(No leer las opciones, esperar que responda y marcar la o las opciones que se asemejen a su respuesta)**

| Respuesta | Sí Menciona |
| --- | --- |
| Una o más porciones de verduras y frutas. |  |
| Agua simple potable a libre demanda. |  |
| Una porción de alimento preparado que cumpla con los criterios establecidos. |  |
| Botanas dulces o saladas solo 1 vez a la semana. |  |
| Leche semidescremada o descremada, yogurt, jugos o néctares hasta 2 veces a la semana. |  |
| No sabe. |  |
| Otra (especificar): ___________________________________________ |  |

1. ¿Hay alimentos que no se venden todos los días? **(No leer las opciones, esperar que responda y marcar la respuesta)**
2. Sí
3. No **(pase a 20)**
4. No sabe **(pase a 20)**
5. ¿Qué alimentos son los que NO se venden todos los días? **(No leer las opciones, esperar que responda y marcar la o las opciones que se asemejen a su respuesta)**
6. Verduras y frutas ¿cuántos días NO se venden? _____
7. Alimentos preparados ¿cuántos días NO se venden? _____
8. Alimentos líquidos permitidos *(leche descremada, leche semidescremada, yogurt, lácteos fermentados, de soya, jugos y néctares)*  ¿cuántos días NO se venden? _____
9. Leche entera ¿cuántos días NO se venden? _____
10. Bebidas para secundaria ¿cuántos días NO se venden? _____
11. Agua embotellada ¿cuántos días NO se venden? _____
12. Botanas dulces ¿cuántos días NO se venden? _____
13. Botanas saladas ¿cuántos días NO se venden? _____
14. Otros (especificar nombre del producto) ¿cuántos días NO se venden? ________
15. ¿En este año, cree usted que la calidad de los alimentos y bebidas que se venden en esta escuela? (Leer las opciones)
16. Ha mejorado
17. Sigue igual
18. Ha empeorado
19. No sabe
20. Por favor, mencione las prácticas higiénicas que se deben seguir en los establecimientos de consumo escolar: (**No leer las opciones, esperar que responda y marcar la o las opciones que se asemejen a su respuesta, acepta más de una opción)**

| Respuesta | Sí Menciona |
| --- | --- |
| Lavarse las manos con agua y jabón antes de preparar alimentos. |  |
| Llevar las uñas cortas y limpias |  |
| Cuidar la higiene de la vestimenta |  |
| Lavar y desinfectar frutas y verduras |  |
| Lavar y desinfectar utensilios de cocina |  |
| Usar cuchillos diferentes para alimentos crudos y cocidos. |  |
| Lavar todos los utensilios antes de preparar alimentos. |  |
| Lavar todos los utensilios después de preparar alimentos. |  |
| Que no haya animales domésticos en el establecimiento. |  |
| Que no haya plagas en el establecimiento. |  |
| Que el lugar donde se ofrecen los alimentos se encuentra alejado de fuentes de contaminación (por ejemplo basura, drenaje abierto, etc.). |  |
| Tapar la basura y eliminarla frecuentemente. |  |
| Que los recipientes y utensilios en donde se encuentran los alimentos estén limpios. |  |
| Mantener los alimentos tapados. |  |
| Refrigerar los alimentos que así lo requieran |  |
| Evitar toser, estornudar o picarse la nariz al preparar o estar en contacto con los alimentos. |  |
| No manejar de manera simultánea el dinero y los alimentos o utilizar guantes o una bolsa de plástico cuando se maneja dinero. |  |
| Cuidar la limpieza de los trapos de cocina. |  |
| Usar trapos de cocina diferentes por cada actividad. |  |
| Usar red para el cabello o gorra al preparar y manipular los alimentos. |  |
| Otra (especifique): |  |

**INFORMACION SOBRE EL FUNCIONAMIENTO DEL COMITÉ DE ESTABLECIMIENTOS DE CONSUMO ESCOLAR**

1. ¿Quiénes conforman el Comité de Establecimientos de Consumo Escolar?

|  | Se identificó a: | ¿Cuántos son? |
| --- | --- | --- |
| Director |  |  |
| Padres y madres o tutores de familia |  |  |
| Personal de apoyo y asistencia a la educación |  |  |
| Profesores |  |  |
| Alumnos |  |  |
| Otro (especificar) ___________________ |  |  |

1. ¿Cuántas veces se ha reunido el Comité de Establecimientos de Consumo Escolar durante este periodo escolar? (**No leer las opciones, esperar que responda y marcar la opción)**
2. No se ha reunido
3. De 1-3 veces
4. 4 a 5 veces
5. 6 veces o más
6. No sabe
7. No aplica
8. ¿Cuáles son las **funciones** del Comité de Establecimientos de Consumo Escolar? (**No leer las opciones, esperar que responda y marcar la o las opciones que se asemejen a su respuesta)**
9. Contribuir en **la promoción de entornos** seguros y saludables mediante la **implementación de prácticas de higiene** en el establecimiento escolar.
10. **Supervisar** que el establecimiento de consumo escolar ofrezca a los alumnos **refrigerios escolares que cumplan con los criterios nutrimentales** establecidos en los Lineamientos generales para el expendio o distribución de alimentos y bebidas de los establecimientos de consumo escolar.
11. **Coordinarse** con el personal de la escuela para promover **una nueva cultura** para el cuidado de la salud de los escolares.
12. **Supervisar la aplicación** sistemática de los Lineamientos generales para el expendio o distribución de los alimentos y bebidas en el establecimiento de consumo escolar.
13. **Supervisar y vigilar** permanentemente la **calidad** de los alimentos y bebidas que se expenden en el establecimiento de consumo escolar (higiene, costo, orden y seguridad).
14. **Propiciar la colaboración** de los padres de familia o tutores y sus asociaciones con el resto de la comunidad educativa, del sector salud **para el desarrollo de acciones de capacitación** a la comunidad escolar para el favorecimiento de una alimentación correcta dentro y fuera de la escuela.
15. **Promover** el consumo de **agua simple** potable
16. No sabe
17. Otro (especificar): ____________
18. ¿Me podría mencionar las estrategias que aplica usted en calidad de responsable del Comité de Establecimiento de Consumo escolar? (**No leer las opciones, esperar que responda y marcar la o las opciones que se asemejen a su respuesta, acepta más de una opción)**
19. Difusión y promoción de la alimentación correcta en el aula, en la escuela y en el hogar
20. Sensibilización, capacitación y asesoría
21. Diseño de materiales de apoyo (carteles, trípticos, folletos, etc.)
22. Diseño e implementación del programa de trabajo
23. Supervisión y seguimiento a la aplicación de las estrategias y acciones
24. Otras (especificar)
25. ¿Piensa Ud. que se podría mejorar el desempeño?

SI |___|

No |___| **(Pase a pregunta 28)**

No sabe |___|

1. ¿Con el apoyo de quién? (**No leer las opciones, esperar que responda y marcar la o las opciones que se asemejen a su respuesta, acepta más de una opción)**
2. Responsable de Establecimientos de Consumo Escolar
3. El director
4. Los miembros del CEPS
5. La comunidad escolar no CEPS
6. No sabe
7. Otro (especificar) ______________

**INFORMACION SOBRE LA UTILIZACION DE LOS MATERIALES PARA LOS ESTABLECIMIENTOS DE CONSUMO ESCOLAR**

| **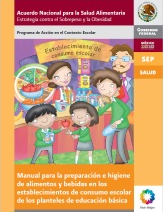**  **A** | 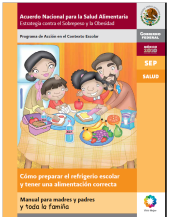 **B** | **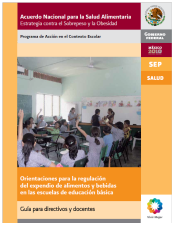**  **C** |
| --- | --- | --- |
| 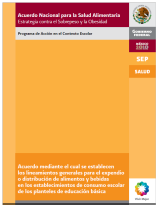  **D** | **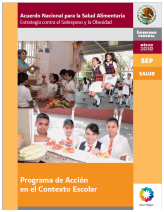**  **E** | **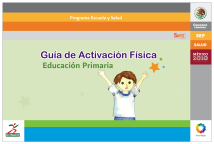**  **F** |
| 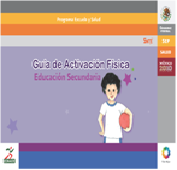  **G** |  |  |

1. ¿Usted **conoce** algunos de estos materiales? **(enseñar cada uno de los materiales y marcar la respuesta correspondiente**)
2. Manual para la preparación e higiene de alimentos y bebidas en los establecimientos de consumo escolar de los planteles de educación básica
3. Cómo preparar el refrigerio escolar y tener una alimentación correcta
4. Orientaciones para la regulación del expendio de alimentos y bebidas en las escuelas de educación básica.
5. Acuerdo mediante el cual se establecen los lineamientos generales para el expendio o distribución de alimentos y bebidas en los establecimientos de consumo escolar de los planteles de educación básica
6. Programa de Acción en el Contexto Escolar
7. Otro (especificar)________________________________________

**En caso de que no conocen ninguno documento, ir a la pregunta 33.**

1. ¿Con qué materiales educativos **cuenta** el Comité de establecimiento de consumo escolar para cumplir con los lineamientos generales para el expendio o distribución de alimentos y bebidas y para el impulso de la activación física? (**No leer las opciones, esperar que responda y marcar la o las opciones que se asemejen a su respuesta)**
2. Manual para la preparación e higiene de alimentos y bebidas en los establecimientos de consumo escolar de los planteles de educación básica
3. Cómo preparar el refrigerio escolar y tener una alimentación correcta
4. Orientaciones para la regulación del expendio de alimentos y bebidas en las escuelas de educación básica.
5. Acuerdo mediante el cual se establecen los lineamientos generales para el expendio o distribución de alimentos y bebidas en los establecimientos de consumo escolar de los planteles de educación básica
6. Programa de Acción en el Contexto Escolar
7. Otro (especificar)________________________________________
8. ¿Cuál de los siguientes materiales ha **consultado**? **(Mostrar las imágenes y marcar cuál o cuáles)**
9. |___|
10. |___|
11. |___|
12. |___|
13. |___|
14. Otro (especificar)________________________________________

**En caso de que no haya consultado ningún material, pasar a la pregunta 33**

1. ¿Qué tan **útiles** han sido los materiales para **la implementación de los lineamientos** generales para el expendio o distribución de alimentos y bebidas?
2. Muy útil
3. Útil
4. Poco útil (“más o menos”)
5. Nada útil
6. No sabe o no los conoce
7. No aplica (No los revisó)
8. ¿Qué tan **útil** han sido los materiales para comprender el **funcionamiento** del Comité de establecimiento de consumo escolar? (**No leer las opciones, esperar que responda y marcar la opción que se asemeje a su respuesta)**
9. Muy útil
10. Útil
11. Poco útil (más o menos)
12. Nada útil
13. No sabe o no los conoce
14. No aplica (No los revisó)

**CONDICIONES QUE DIFICULTAN O FACILITAN LAS TAREAS DEL COMITÉ DE ESTABLECIMIENTOS DE CONSUMO ESCOLAR**

1. ¿Qué tan de acuerdo está en que se ha logrado cumplir con las directivas de los lineamientos generales para el expendio o distribución de alimentos y bebidas? (**No leer las opciones, esperar que responda y marcar la opción que se asemeje a su respuesta)**
2. Totalmente de acuerdo
3. De acuerdo
4. Ni de acuerdo ni en desacuerdo (indiferente)
5. En desacuerdo
6. Totalmente en desacuerdo
7. No sabe
8. Otro (especificar)____________________
9. De manera general, ¿Cuáles considera que son, en su escuela, los principales obstáculos o barreras para el cumplimiento de los lineamientos generales para el expendio o distribución de alimentos y bebidas? **(No leer las opciones, esperar que responda y marcar la o las opciones que se asemeje a su respuesta)**
10. Desinformación o desconocimiento de los lineamientos
11. Medidas poco adaptadas a la situación local
12. Poca supervisión a las escuelas por parte de las autoridades correspondientes
13. Convenios entre empresas y gobierno (poca credibilidad y contradicción)
14. Venta clandestina por parte de los niños o profesores
15. Presencia de puestos ambulantes afuera de escuela que no acatan los lineamientos
16. Poco interés e involucramiento de padres
17. Preferencia de los niños hacia la comida chatarra
18. Resistencia al cambio de los profesores y/o directores
19. Hábitos/costumbres familiares poco saludables
20. Limitantes económicas
21. Influencia del marketing de alimentos y bebidas dirigida a los niños
22. Falta de apoyo por parte de las autoridades educativas
23. Falta de apoyo de las autoridades de salud
24. Falta de capacitación
25. Falta de materiales de apoyo
26. Otro (especificar) ___________________________________________________
27. ¿Cuáles considera que son las condiciones que facilitan la implementación los lineamientos generales para el expendio o distribución de alimentos y bebidas? **(No leer las opciones, esperar que responda y marcar la opción que se asemeje a su respuesta)**
28. Entusiasmo y disposición de profesores
29. Responsabilidad/liderazgo de los directores
30. Reconocimiento de la comunidad escolar de ser una escuela transmisora de valores de auto cuidado y salud
31. Apoyo de los padres y madres de familia/ tutores
32. Apoyo por parte de los supervisores de la zona escolar
33. Participación activa de los comités
34. Capacitación y asesoría
35. Disponibilidad de materiales
36. Apoyo de autoridades educativas
37. Apoyo de autoridades de salud
38. Participación del Comité de establecimientos de consumo escolar
39. Otro (especificar)______

**¡Muchas gracias por su participación!**

1. **Questionnaire with PAF Members**

**INSTITUTO NACIONAL DE SALUD PÚBLICA**

**CENTRO DE INVESTIGACIÓN EN NUTRICIÓN Y SALUD**

**ESTUDIO SOBRE LA APLICACIÓN DE LA ETAPA II DE LOS LINEAMIENTOS GENERALES PARA EL EXPENDIO O DISTRIBUCIÓN DE ALIMENTOS Y BEBIDAS EN LOS ESTABLECIMIENTOS DE CONSUMO ESCOLAR DE LOS PLANTELES DE EDUCACIÓN BÁSICA**

**CUESTIONARIO PARA INTEGRANTES DEL COMITÉ DE IMPULSO A LA ACTIVACION FISICA**

**FICHA DE IDENTIFICACIÓN**

1. Código del entrevistador |__|__| 2. Código de la escuela |__|__|

3. Clave de la escuela |__|__| 4. Localidad |__|__|

5. Municipio |__|__| 6. Estado |__|__|

7. Nombre de la escuela: __________________________________________________________

8. Turno: ______________________

9. Dirección de la escuela: ________________________________________________________________________________________________________________________________________________________________

10. Código del formato: |__|__|__|

11. Código del encuestado: |__|__|__|__|__|__|

1. Fecha de aplicación del cuestionario |__|__|__|__|__|__|

Día Mes Año

1. Usted es integrante o cubre las funciones de :

CEPS |___| (1. Si, 2.No, NA cuando no hay CEPS)

Comité de Establecimientos de Consumo Escolar |___| (1. Si, 2.No, NA cuando no hay Comité de Establecimiento)

Comité de Impulso de la Activación física |___| (1. Si, 2.No, NA cuando no hay Comité de Impulso)

1. ¿Desde cuándo? (**Poner duración en meses o NA**)

CEPS |___|

Comité de Establecimientos |___|

Comité de Impulso |___|

1. Usted está involucrado en calidad de :

Profesor de grupo |___| (1. Si, 2.No, o NA)

Profesor de educación física |___| (1. Si, 2.No, o NA)

Madre/padre de familia o tutor |___| (1. Si, 2.No, o NA)

Otro (**especificar**) _______________

1. Pertenencia a la asociación de padres de familia de la escuela **|___|(solamente para madre/padre de familia)**
2. Nivel de escolaridad |___|
3. Sexo |___| (1. Mujer, 2. Hombre)
4. Edad |___|___| (años)

**INFORMACIÓN RELATIVA A LA APLICACIÓN DE LA ETAPA II DE LOS LINEAMIENTOS GENERALES PARA EL EXPENDIO O DISTRIBUCIÓN DE ALIMENTOS Y BEBIDAS EN LOS ESTABLECIMIENTOS DE CONSUMO ESCOLAR DE LOS PLANTELES DE EDUCACIÓN BÁSICA**

1. ¿Conoce Usted los lineamientos generales para el expendio o distribución de alimentos y bebidas los establecimientos de consumo escolar de los planteles de educación básica?
   1. Sí
   2. No

**Leer la siguiente oración de explicación:**

Los lineamientos son un conjunto de acciones de la SEP sobre la venta de alimentos y la realización de actividad física en la escuela.

1. ¿Conoce Usted qué propició la creación de estos lineamientos generales para el expendio o distribución de alimentos y bebidas? **(No leer las opciones, esperar que responda y marcar la o las opciones que se asemejen a su respuesta, acepta más de una opción)**
2. Sobrepeso/**obesidad**
3. Diabetes
4. Sedentarismo
5. Falta de disponibilidad de agua simple potable
6. Amplia disponibilidad de alimentos con alto contenido calórico
7. No sabe
8. Otro (especificar)_____________________________________________________
9. ¿En qué consiste la aplicación de los lineamientos generales para el expendio o distribución de alimentos y bebidas? **(No leer las opciones, esperar que responda y marcar la o las opciones que se asemejen a su respuesta)**
10. Regular la venta de alimentos y bebidas dentro de la escuela (o cualquier opción relacionada solo con alimentación y nutrición).
11. Mejorar la práctica de actividad en las escuelas (o cualquier opción relacionada sólo con activación física).
12. Promover un refrigerio saludable proporcionando información a los padres.
13. Privilegiar la disponibilidad y consumo de agua potable
14. No sabe
15. Otro (especificar)_____________________________________________________
16. ¿La comunidad educativa ha recabado recursos económicos para la aplicación de los lineamientos generales para el expendio o distribución de alimentos y bebidas?
    1. Sí
    2. No
    3. No sabe
17. ¿La aplicación de los Lineamientos generales para el expendio o distribución de alimentos y bebidas implicó algún cambio para su escuela? **(No leer las opciones, esperar que responda y marcar la respuesta)**
    1. Sí
    2. No **(pase a la pregunta 7)**
    3. No sabe **(pase a la pregunta 7)**
18. ¿Cuáles fueron los cambios? **(No leer las opciones, esperar que responda y marcar la o las opciones que se asemejen a su respuesta)**
19. Cambios relacionados con la venta de alimentos
20. Cambios relacionados con la venta de bebidas
21. Cambios relacionados con la disponibilidad de agua simple potable
22. Cambios relacionados con la activación física regular
23. Cambios relacionados con la promoción de una alimentación correcta
24. Cambios relacionados con la promoción de activación física regular
25. Cambios relacionados con el incremento de la participación del comité
26. Otro (especificar) _____________________________
27. ¿Podría mencionar en qué etapa de aplicación de los Lineamientos generales para el expendio o distribución de alimentos y bebidas nos encontramos en este ciclo escolar? ? (**No leer las opciones, esperar que responda y marcar la o las opciones que se asemejen a su respuesta**)
28. Etapa I
29. Etapa II
30. Etapa III
31. No sabe
32. Otro (especificar)
33. ¿Conoce Usted los cambios que diferencian la etapa I de la II de los lineamientos generales para el expendio o distribución de alimentos y bebidas? **(No leer las opciones, esperar que responda y marcar la respuesta)**
34. Sí
35. No **(pase a la pregunta 10)**
36. ¿Cuáles son? **(No leer las opciones, esperar que responda y marcar la o las opciones que se asemejen a su respuesta)**
    1. Disminución de **grasas, sales y azúcares** en los productos que se expenden en los establecimientos de consumo escolar
    2. Se **prioriza** la venta de **alimentos preparados**
    3. **Disminución de porciones** en los alimentos que se expenden
    4. **Eliminación** de la venta de algunos productos industrializados
    5. **Aumento** de consumo de **verduras y frutas**
    6. Otro. (Especificar)________________________________
37. Dentro de la escuela ¿Alguna persona o personas supervisan la aplicación de los lineamientos generales para el expendio o distribución de alimentos y bebidas? **(No leer las opciones, esperar que responda y marcar la respuesta)**
    1. Sí
    2. No (**pase a la pregunta 12)**
    3. No sabe (**pase a la pregunta 12)**
38. ¿Quién supervisa el cumplimiento? (**No leer las opciones, esperar que responda y marcar la o las opciones que se asemejen a su respuesta)**
39. Director
40. Profesores
41. Asociación de padres de familia
42. Supervisor de la Zona Escolar
43. Integrante(s) del Comité de Establecimiento al Consumo Escolar
44. Otro (especificar):

**FUNCIONAMIENTO DEL COMITÉ DE IMPULSO A LA ACTIVACION FISICA**

1. ¿Quiénes conforman el Comité de impulso a la activación física?

|  | Se identificó a: | ¿Cuántos son? |
| --- | --- | --- |
| Director |  |  |
| Padres y madres o tutores de familia |  |  |
| Personal de apoyo y asistencia a la educación |  |  |
| Profesores |  |  |
| Alumnos |  |  |
| Otro (especificar) ___________________ |  |  |

1. ¿Cuántas veces se ha reunido el Comité de Impulso a la Activación Física durante este ciclo escolar? **(No leer las opciones, esperar que responda y marcar la opción que se asemeje a su respuesta)**
2. No se ha reunido
3. De 1-3 veces
4. 4 a 5 veces
5. 6 veces o más
6. No sabe
7. No aplica
8. ¿Se realiza Activación Física regular en la escuela? (**No leer las opciones, esperar que responda y marcar la respuesta)**
9. Sí
10. No **(pase pregunta 16)**
11. ¿En qué momento dentro del horario escolar se realiza la activación física?

| **Tipo de actividad** | **Número de días a la semana** | **Lugar en dónde la realizan** | **¿Quién la dirige?** | **¿Cuánto dura?** |
| --- | --- | --- | --- | --- |
| Activación al arrancar la jornada escolar |  |  |  |  |
| Activación al final de la jornada |  |  |  |  |
| Activación dentro del salón de clase |  |  |  |  |
| Recreo activo |  |  |  |  |
| Otro ( especificar) |  |  |  |  |

1. ¿Cuáles son las **funciones** del comité de impulso a la activación física? **(No leer las opciones, esperar que responda y marcar la o las opciones que se asemejen a su respuesta)**
2. Incentivar la implementación y uso de las Guías de Activación Física para profesores de educación básica.
3. Promover la recuperación de espacios seguros en la escuela y en lugares cercanos a ella, para el impulso de la activación física regular.
4. Buscar recursos de apoyo, personal especializado e insumos básicos para la implementación de la activación física regular en la escuela.
5. Gestionar acciones y apoyos con instancias externas a la escuela para favorecer la activación física.
6. Otro (especificar): ________________
7. No sabe
8. ¿Me podría mencionar las **estrategias** que utiliza para impulsar la activación física dentro de la escuela? **(esperar que responda y marcar la o las opciones que se asemejen a su respuesta)**
9. Difusión y promoción de la activación física, en el aula, en la escuela y en el hogar
10. Sensibilización, capacitación y asesoría
11. Diseño de materiales de apoyo (carteles, trípticos, folletos, películas, etc.)
12. Diseño e implementación del programa de trabajo
13. Seguimiento y evaluación a la aplicación de las estrategias y acciones
14. Otras (especificar)

1. ¿Piensa Ud., que se podría mejorar el desempeño del Comité de Impulso a la Activación física?

SI |___|

No |___| **(pase a 20)**

No sabe |___|

1. ¿Con el apoyo de quién? **(Esperar que responda y marcar la o las opciones que se asemejen a su respuesta)**
2. El director |___|
3. Profesores de educación física |___|
4. Profesores de grupo |___|
5. Padres de familia
6. Otro (especificar) ______________

**INFORMACION SOBRE LA UTILIZACION DE LOS MATERIALES PARA EL IMPULSO DE LA ACTIVACION FISICA**

MATERIAL

| **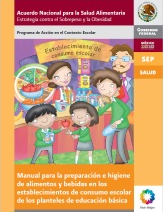**  **A** | 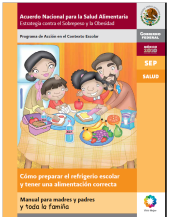 **B** | **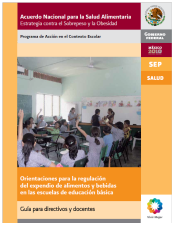**  **C** |
| --- | --- | --- |
| 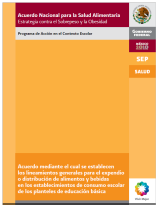  **D** | **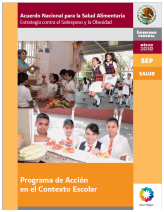**  **E** | **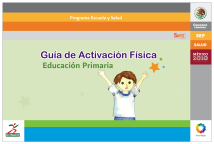**  **F** |
| 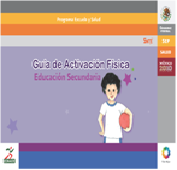  **G** |  |  |

1. ¿Usted **conoce** algunos de estos materiales? **(enseñar cada uno de los materiales y marcar la respuesta correspondiente**)
2. Programa de Acción en el Contexto Escolar
3. Acuerdo mediante el cual se establecen los lineamientos generales para el expendio o distribución de alimentos y bebidas en los establecimientos de consumo escolar de los planteles de educación básica
4. Guías de activación física (guía primaria)
5. Guías de activación física (guía secundaria)
6. Otro

**En caso de que no conocen ninguno documento, ir a la pregunta 25.**

1. ¿Con qué materiales educativos **cuenta** el Comité de impulso a la activación física para el impulso de la activación física regular? **(No leer las opciones, esperar que responda y marcar la o las opciones que se asemejen a su respuesta)**
2. Programa de Acción en el Contexto Escolar
3. Acuerdo mediante el cual se establecen los lineamientos generales para el expendio o distribución de alimentos y bebidas en los establecimientos de consumo escolar de los planteles de educación básica
4. Guías de activación física (guía primaria)
5. Guías de activación física (guía secundaria)
6. Otro (especificar)________________________________________
7. ¿Cuál de los siguientes materiales ha **consultado**? **(Mostrar las imágenes y marcar cuál o cuáles)**

D |___|

E |___|

F |___| (Solamente para primaria)

G |___| (Solamente para secundaria)

**En caso de que no haya consultado ningún material, pasar a la pregunta 25.**

1. ¿Qué tan útiles han sido los materiales para el **impulso** de la activación física?
2. Muy útil
3. Útil
4. Poco útil (“más o menos”)
5. Nada útil
6. No sabe o no los conoce
7. No aplica (No los revisó)
8. ¿Qué tan útiles han sido los materiales para comprender el **funcionamiento** del Comité de impulso a la activación física? (**No leer las opciones, esperar que responda y marcar la opción que se asemeje a su respuesta)**
9. Muy útil
10. Útil
11. Poco útil (“más o menos”)
12. Nada útil
13. No sabe o no los conoce
14. No aplica (No los revisó)

**CONDICIONES QUE DIFICULTAN O FACILITAN EL IMPULSO A LA ACTIVACIÓN FÍSICA**

1. ¿Qué tan de acuerdo está en que se ha logrado cumplir con las directivas de los lineamientos generales para el impulso de la activación física dentro de su escuela? (**No leer las opciones, esperar que responda y marcar la opción que se asemeje a su respuesta)**
2. Totalmente de acuerdo
3. De acuerdo
4. Ni de acuerdo ni en desacuerdo (indiferente)
5. En desacuerdo
6. Totalmente en desacuerdo
7. No sabe
8. Otro (especificar)____________________
9. De manera general, ¿Cuáles considera que son **los principales obstáculos o barreras** para la implementación de la activación física en su escuela? (**No leer las opciones, esperar que responda y marcar la o las opciones que se asemejen a su respuesta)**
10. Desinformación o desconocimiento de los Lineamientos en cuanto a la activación física
11. Medidas poco adaptadas a la situación local
12. Poca supervisión a las escuelas por parte de las autoridades correspondientes
13. Falta de profesores de educación física
14. Resistencia de los profesores de grupo en llevar a cabo actividades físicas con sus alumnos
15. Poco interés e involucramiento de padres
16. Resistencia de los niños a hacer activación física regular
17. Resistencia al cambio de los profesores y/o directores
18. Falta de espacio dentro de la escuela
19. Falta de apoyo por parte de las autoridades educativas
20. Falta de apoyo de las autoridades de salud
21. Falta de capacitación y asesoría
22. Otro (especificar) ______
23. ¿Cuáles son los factores que facilitan la implementación de la activación física en su escuela? (**No leer las opciones, esperar que responda y marcar la o las opciones que se asemejen a su respuesta)**
24. Entusiasmo y disposición de profesores
25. Responsabilidad/liderazgo de los directores
26. Reconocimiento de la comunidad escolar de ser una escuela transmisora de valores de auto cuidado y salud
27. Apoyo de los padres y madres de familia/ tutores
28. Apoyo por parte de los supervisores de la zona escolar
29. Disponibilidad de materiales
30. Apoyo de autoridades educativas
31. Apoyo de autoridades de salud
32. Capacitación y asesoría
33. Participación activa de los comités
34. Otro (especificar) ______

**¡Muchas gracias por su participación!**

**3 Questionnaire with Parents** (Excluding the one from committees)

**ESTUDIO SOBRE LA APLICACIÓN DE LA ETAPA II DE LOS LINEAMIENTOS GENERALES PARA EL EXPENDIO O DISTRIBUCIÓN DE ALIMENTOS Y BEBIDAS EN LOS ESTABLECIMIENTOS DE CONSUMO ESCOLAR DE LOS PLANTELES DE EDUCACIÓN BÁSICA**

**CUESTIONARIO PARA PADRES Y MADRES DE FAMILIA**

**FICHA DE IDENTIFICACIÓN**

1. Código del entrevistador |__|__| 2. Código de la escuela |__|__|

3. Clave de la escuela |__|__|__|__||__| 4. Localidad |__|__|

5. Municipio |__|__| 6. Estado |__|__|

7. Nombre de la escuela ____________________________________________

8. Turno: ___________________________

9. Dirección de la escuela: ______________________________________________________________________________________________________________________________________

10. Fecha de aplicación del cuestionario |__|__|__|__|__|__|

Día Mes Año

11. Nombre del padre o madre de familia

___________________________________________________________

1. Edad del padre o madre de familia______
2. Escolaridad del padre (madre) de familia:
3. No sabe leer y escribir
4. Sabe leer y escribir
5. Primaria inconclusa
6. Primaria terminada
7. Secundaria inconclusa
8. Secundaria terminada
9. Bachillerato inconcluso
10. Bachillerato terminado o carrera técnica
11. Licenciatura ó más

**INFORMACIÓN GENERAL DE LA APLICACIÓN DE LOS LINEAMIENTOS SOBRE LA VENTA DE ALIMENTOS EN LA ESCUELA**

1. ¿Conoce usted los lineamientos generales para el expendio o distribución de alimentos y bebidas
2. si
3. no

**Leer la siguiente oración de explicación:**

**“Los lineamientos son un conjunto de acciones de la SEP sobre la venta de alimentos y la realización de actividad física en la escuela”**

1. ¿Sabe qué propició la creación de estos lineamientos? (*No leer las opciones, esperar que responda y marcar la o las opciones que se asemejen a su respuesta).*
2. Sobrepeso/obesidad
3. Diabetes
4. Sedentarismo
5. Falta de disponibilidad de agua simple potable
6. Amplia disponibilidad de alimentos con alto contenido calórico
7. No sabe
8. Otros especificar):____________________________________________
9. ¿En qué consiste la aplicación de los lineamientos generales para el expendio o distribución de alimentos y bebidas? *(No leer las opciones, esperar que responda y marcar la o las opciones que se asemejen a su respuesta).*

1. Regular la venta de alimentos y bebidas dentro de la escuela (o cualquier opción relacionada solo con alimentación y nutrición)
2. Mejorar la práctica de actividad física en las escuelas (o cualquier opción relacionada sólo con activación física).
3. Promover un refrigerio saludable proporcionando información a los padres.
4. Facilitar la disponibilidad y consumo de agua simple potable
5. No sabe (pasar a la pregunta 12)
6. Otro (especificar): ____________________________________________
7. ¿La aplicación de los lineamientos generales para el expendio o distribución de alimentos y bebidas implicó algún cambio en la escuela de su hijo(a)?
   1. Sí (pase a la pregunta 5)
   2. No (pase a la pregunta 6)
8. ¿Sabe usted en que consistieron los cambios en la venta de alimentos en la tienda escolar a partir de que se implementaron los lineamientos? *(No leer las opciones, esperar que responda y marcar la o las opciones que se asemejen a su respuesta)*
9. Cambios relacionados con la venta de alimentos
10. Cambios relacionados con la venta de bebidas
11. Cambios relacionados con la disponibilidad de agua simple potable
12. Cambios relacionados con más realización de activación física regular
13. Cambios relacionados con la promoción de una alimentación correcta
14. Cambios relacionados con la promoción de actividad física regular
15. Cambios relacionados con el incremento de la participación del comité
16. Otro (especificar) __________________________________________
17. ¿Cómo se enteró de los Lineamientos generales para el expendio o distribución de alimentos y bebidas? *(No leer las opciones, esperar que responda y marcar la o las opciones que se asemejen a su respuesta)*
18. A través del Consejo Escolar de Participación Social (CEPS)
19. A través del comité de establecimiento de consumo escolar
20. A través del comité de activación física
21. A través del director
22. A través de los profesores
23. A través de la asociación de padres de familia
24. A través de material impreso
25. Otro (especificar)_____________________________________________
26. ¿Alguna persona o personas supervisan la aplicación de los lineamientos generales para el expendio o distribución de alimentos y bebidas?
27. Sí (pase a la pregunta 8)
28. No (pase a la pregunta 9)
29. No sabe (pase a la pregunta 9)
30. ¿Quién supervisa el cumplimiento? (acepta más de una opción)
31. Director
32. Profesores
33. Padres de familia
34. Supervisor de la zona escolar
35. Integrante(s) del Comité de Establecimiento al Consumo Escolar
36. Otro (especificar)
37. ¿Podría mencionar cuáles son los principales criterios nutrimentales que definen los Lineamientos generales para el expendio o distribución de alimentos y bebidas? *(No leer las opciones, esperar que responda y marcar la o las opciones que se asemejen a su respuesta).*

|  |  |
| --- | --- |
| 1. Conformación de un refrigerio escolar que contribuya a una alimentación saludable para los alumnos. |  |
| 1. Priorizar la venta de verduras y frutas. |  |
| 1. Priorizar la venta de agua simple potable. |  |
| 1. Priorizar la venta de alimentos preparados que facilitan una dieta correcta |  |
| 1. Que las características nutrimentales de los alimentos y bebidas sean de acuerdo a la cantidad de energía y nutrimentos que requieren los estudiantes para promover y mantener su salud. |  |
| 1. Que las bebidas con edulcorantes no calóricos se vendan solo en secundarias |  |
| 1. Que los alimentos cumplan con las normas higiénicas de preparación y consumo |  |
| 1. Que 1 vez por semana, se podrán sustituir las preparaciones del refrigerio por alguna botana(galletas, pastelillos, confites y/o postres) |  |
| 1. Que 2 veces por semana, se podrá sustituir el alimento preparado del refrigerio por alimentos líquidos (leche semidescremada y descremada, yogurt, alimentos líquidos de soya o jugos). |  |

1. ¿En este año, cree usted que la calidad de los alimentos y bebidas que se expenden en esta escuela ha mejorado, sigue igual o ha empeorado?
2. Ha mejorado
3. Sigue igual
4. Ha empeorado
5. No sabe

1. ¿Conoce usted un listado de alimentos y bebidas que cumplen con los criterios nutrimentales de los lineamientos generales y pueden ser expendidos para su venta en las escuelas?
2. Si
3. No
4. ¿Sabe qué alimentos se ofrecen en la tienda escolar? *Leer opciones*

|  | Si o no | Días de la semana |
| --- | --- | --- |
| 1. Verduras y frutas |  |  |
| 1. Alimentos preparados no fritos |  |  |
| 1. Alimentos preparados fritos |  |  |
| 1. Alimentos industrializados no fritos (botanas no fritas) |  |  |
| 1. Alimentos industrializados fritos (botanas fritas) |  |  |
| 1. Dulces, Chicles y confites |  |  |

1. ¿Sabe qué alimentos o bebidas líquidos se ofrecen en la tienda escolar?

|  | Si o no | Días de la semana |
| --- | --- | --- |
| 1. Leche entera |  |  |
| 1. Leche descremada o semidescremada |  |  |
| 1. Yogurt |  |  |
| 1. Jugos de frutas |  |  |
| 1. Néctares |  |  |
| 1. Jugos de verduras |  |  |
| 1. Líquidos de soya |  |  |
| 1. Alimento lácteo fermentado |  |  |
| 1. Refrescos y bebidas azucaradas |  |  |
| 1. agua simple potable |  |  |

1. ¿Sabe usted qué elementos debe incluir un refrigerio escolar saludable? *(No leer las opciones, esperar que responda y marcar la o las opciones que se asemejen a su respuesta)*
2. Una o más porciones de verduras y frutas
3. Agua simple potable a libre demanda
4. Una porción de alimento preparado que cumpla con los criterios nutrimentales establecidos
5. Botanas dulces o saladas solo 1 vez a la semana
6. Leche semidescremada, descremada, yogurt, alimentos líquidos de soya, jugos o néctares hasta 2 veces a la semana
7. leche entera
8. bebidas azucaradas o refrescos
9. No sabe
10. Otra (especificar):_____________________________________________

INFORMACIÓN SOBRE UTILIZACION DE LOS MATERIALES

1. ¿Le entregaron algún folleto o cuadernillo u otro material impreso donde le hicieran saber en qué consisten los lineamientos generales para el expendio o distribución de alimentos y bebidas?
2. si
3. no (pase a la pregunta 18).
4. ¿Puede identificar el material impreso le entregaron? *(mostrar el material y marcar el material que el padre de familia refiera).*
5. Manual para padres y madres de familia “Como preparar el refrigerio escolar y tener una alimentación correcta.
6. otro
7. ¿Qué tan útiles han sido los materiales para la implementación de los lineamientos generales para el expendio o distribución de alimentos y bebidas?
8. Muy útil
9. Útil
10. Poco útil (más o menos)
11. Nada útil
12. No sabe o no los conoce
13. No aplica (No los revisó)

**CONDICIONES QUE DIFICULTAN O FACILITAN LA APLICACIÓN DE LOS LINEAMIENTOS GENERALES DE PARA EL EXPENDIO O DISTRIBUCIÓN DE ALIMENTOS Y BEBIDAS EN LOS ESTABLECIMIENTOS DEL CONSUMO ESCOLAR**

1. ¿Qué tan de acuerdo está en que se ha logrado cumplir con los lineamientos generales para el expendio o distribución de alimentos y bebidas en la escuela de su hijo?
2. Totalmente de acuerdo
3. De acuerdo
4. Ni de acuerdo ni en desacuerdo (indiferente)
5. En desacuerdo
6. Totalmente en desacuerdo
7. No sabe
8. Otro (especificar)
9. De manera general, ¿Cuáles considera que son, en la escuela de su hijo, los principales obstáculos o barreras para el cumplimiento de los lineamientos generales para el expendio o distribución de alimentos y bebidas? (*No leer las opciones, esperar que responda y marcar la o las opciones que se asemejen a su respuesta)*
10. Desinformación o desconocimiento de los lineamientos
11. Medidas poco adaptadas a la situación local
12. Poca supervisión a las escuelas por parte de las autoridades correspondientes
13. Convenios entre empresas y gobierno (poca credibilidad y contradicción)
14. Venta clandestina por parte de los niños o profesores
15. Presencia de puestos ambulantes afuera de escuela que no acatan los lineamientos
16. Poco interés e involucramiento de padres
17. Preferencia de los niños hacia la comida no saludable (chatarra)
18. Resistencia de los profesores y/o directores
19. Hábitos/costumbres familiares poco saludables
20. Limitantes económicas
21. Influencia del marketing de alimentos y bebidas dirigida a los niños
22. Falta de apoyo por parte de las autoridades educativas
23. Falta de apoyo de las autoridades de salud
24. Falta de capacitación
25. Falta de materiales de apoyo
26. Otro (especificar)_______________________________________
27. ¿Cuáles considera que son las condiciones que facilitan la implementación de los lineamientos generales para el expendio o distribución de alimentos y bebidas?  **(***No leer las opciones, esperar que responda y marcar la o las opciones que se asemejen a su respuesta)*
28. Entusiasmo y disposición de profesores
29. Responsabilidad/liderazgo de los directores
30. Reconocimiento de la comunidad escolar de ser una escuela transmisora de valores de auto cuidado y salud
31. Apoyo de los padres y madres de familia/ tutores
32. Apoyo por parte de los supervisores de la Zona Escolar
33. Participación activa de los Comités
34. Capacitación y asesoría
35. Disponibilidad de materiales
36. Apoyo de autoridades educativas
37. Apoyo de autoridades de salud
38. Participación del Comité de establecimientos de consumo escolar
39. Otro (especificar)______

**INFORMACIÓN SOBRE LA ACTIVACIÓN FÍSICA**

1. ¿Se realiza Activación Física regular en la escuela de su hijo (además de las clases de educación física)?
2. Sí
3. no (pase a la pregunta 26)
4. no sabe (pase a la pregunta 26)
5. ¿En qué momento dentro del horario escolar se realiza la activación física?

| **Tipo de actividad** | **Número de días a la semana** | **Lugar en dónde la realizan** | **¿Quién la dirige?** | **¿Cuánto dura?** |
| --- | --- | --- | --- | --- |
| 1. Activación al arrancar la jornada escolar |  |  |  |  |
| 1. Activación dentro del salón de clase |  |  |  |  |
| 1. Activación al final de la jornada |  |  |  |  |
| 1. Recreo activo |  |  |  |  |
| 1. Otro (especificar) |  |  |  |  |

**CONDICIONES QUE DIFICULTAN O FACILITAN LA APLICACIÓN DE LOS LINEAMIENTOS EN EL IMPULSO DE LA ACTIVACION FISICA**

1. ¿Qué tan de acuerdo está en que se ha logrado cumplir con los lineamientos en su componente de activación física dentro de la escuela de su hijo?

1. Totalmente de acuerdo
2. De acuerdo
3. Ni de acuerdo ni en desacuerdo (indiferente)
4. En desacuerdo
5. Totalmente en desacuerdo
6. No sabe
7. Otro (especificar)____________________
8. ¿Cuáles considera que son los principales obstáculos o barreras para la implementación de la activación física en la escuela de su hijo? (*No leer las opciones, esperar que responda y marcar la o las opciones que se asemejen a su respuesta)*
9. Desinformación o desconocimiento de los lineamientos en cuanto a la activación física)
10. Medidas poco adaptadas a la situación local
11. Poca supervisión a las escuelas por parte de las autoridades correspondientes
12. Falta de profesores de educación física
13. Resistencia de los profesores de grupo en llevar a cabo a actividades físicas con sus alumnos
14. Poco interés e involucramiento de padres
15. Resistencia de los niños a hacer activación física regular
16. Resistencia al cambio de los profesores y/o directores
17. Falta de espacios dentro de la escuela
18. Falta de apoyo por parte de las autoridades educativas
19. Falta de apoyo de las autoridades de salud
20. Falta de capacitación y asesoría
21. Otro (especificar) ______
22. ¿Qué factores considera usted que facilitan la implementación de la activación física en la escuela de su hijo?
23. Entusiasmo y disposición de profesores
24. Responsabilidad/liderazgo de los directores
25. Reconocimiento de la comunidad escolar de ser una escuela transmisora de valores de auto cuidado y salud
26. Apoyo de los padres y madres de familia/ tutores
27. Apoyo por parte de los supervisores de la Zona Escolar
28. Disponibilidad de materiales
29. Apoyo de autoridades educativas
30. Apoyo de autoridades de salud
31. Capacitación y asesoría
32. Participación activa de los comités
33. Otro (especificar) ______

**INFORMACIÓN GENERAL DEL REFRIGERIO QUE PROPORCIONAN LOS PADRES A SUS HIJOS**

26. ¿Su hijo toma algún alimento antes de acudir a la escuela?

a) si

b) no

27. ¿Normalmente su niño acude con refrigerio/lunch a la escuela?

a) si

b) no (pase a la pregunta 32)

1. ¿Qué alimentos contiene el refrigerio/lunch de su hijo? (*No leer las opciones, esperar que responda y marcar la o las opciones que se asemejen a su respuesta).*
2. Fruta y/o verdura
3. Agua simple potable
4. Bebida azucarada o refresco
5. Lácteos/yogurt
6. Alimentos preparados no fritos
7. Alimentos preparados fritos o comida rápida
8. Botanas dulces (pastelillos, galletas, chocolates, dulces, confites)
9. Botanas saladas
10. Barras de cereal
11. Otros (especificar)
12. A partir de la aplicación de los Lineamientos ¿ha realizado usted algún cambio en los alimentos que conforman el refrigerio escolar de su hijo?

a) si

b) no (pase a la pregunta 32)

1. ¿Qué cambios ha realizado en los alimentos que conforman el refrigerio escolar de su hijo? (*No leer las opciones, esperar que responda y marcar la o las opciones que se asemejen a su respuesta).*
2. Inclusión de agua simple potable
3. Inclusión de una o más porciones de verduras o frutas
4. Inclusión de una porción de alimento preparado que cumpla con los criterios nutrimentales establecidos
5. Inclusión de leche semidescremada, descremada, yogurt, alimentos líquidos de soya, jugos o néctares hasta 2 veces a la semana
6. Eliminación de botanas dulces o saladas
7. Eliminación de alimentos fritos preparados
8. Eliminación de leche entera
9. Eliminación de bebidas azucaradas o refrescos

1. ¿Usted da dinero a su hijo para comprar alimentos dentro de la escuela?

a) si

b) no (pase a la pregunta 34).

1. ¿Cuántos días de la semana usted da dinero a su hijo para comprar alimentos dentro de la escuela? (*No leer las opciones, esperar que responda y marcar la o las opciones que se asemejen a su respuesta).*
2. Un día
3. Dos días
4. Tres días
5. Cuatro días
6. Todos los días
7. Cada vez que da dinero a su hijo ¿Cuánto dinero le da para comprar alimentos dentro de la escuela?

1. Cinco pesos o menos
2. entre 6 y 10 pesos
3. Entre 11 y 15 pesos
4. Entre 16 y 20 pesos
5. Entre 21 y 30 pesos
6. Más de 30 pesos

**CARTILLA NACIONAL DE SALUD**

1. ¿A usted o a su(s) hijo(s) le entregaron la Cartilla Nacional de Salud en la escuela?

a) si

b) no (finalice el cuestionario)

c) No sabe o no lo recuerda (finalice el cuestionario)

1. ¿Quien le entrego la cartilla Nacional de Salud de su hijo?
2. El director de la escuela
3. el profesor del grupo al que pertenece su hijo
4. Se la enviaron con su hijo
5. otro (especificar) ___________________
6. ¿Le explicaron cual es el uso de la Cartilla Nacional de Salud?
7. si
8. no
9. ¿Acudió con la cartilla Nacional de su hijo al Centro de Salud u otras instituciones de Salud (IMSS, ISSSTE)?
10. si
11. no

1. ¿Actualmente utiliza la cartilla Nacional de Salud de su hijo?
2. si
3. no

**4. Questionnaire with teachers** (Excluding the ones from committees)

**INSTITUTO NACIONAL DE SALUD PÚBLICA**

**CENTRO DE INVESTIGACIÓN EN NUTRICIÓN Y SALUD**

**ESTUDIO SOBRE LA APLICACIÓN DE LA ETAPA II DE LOS LINEAMIENTOS GENERALES PARA EL EXPENDIO O DISTRIBUCIÓN DE ALIMENTOS Y BEBIDAS EN LOS ESTABLECIMIENTOS DE CONSUMO ESCOLAR DE LOS PLANTELES DE EDUCACIÓN BÁSICA**

**CUESTIONARIO PARA PROFESORES**

**FICHA DE IDENTIFICACIÓN**

1. Código del entrevistador |__|__| 2. Código de la escuela |__|__|

3. Clave de la escuela |__|__| 4. Localidad |__|__|

5. Municipio |__|__| 6. Estado |__|__|

7. Nombre de la escuela ____________________________________________

8. Turno: ______________________

9. Dirección de la escuela: ________________________________________________________________________________________________________________________________________________________________

10. Código de formato: |__|__|__|

11. Código del encuestado: |__|__|__|__|__|__|

12. Fecha de aplicación del cuestionario |__|__|__|__|__|__|

Día Mes Año

1. Nombre del profesor: ______________________________________________
2. Sexo (1. Mujer, 2. Hombre)
3. Edad (años)
4. Grado/Grupo |___|___| |___|___|
5. Años de docencia
6. Años de docencia en esta escuela
7. ¿Imparte clases en otra escuela?
   1. Sí
   2. No

**INFORMACIÓN RELATIVA A LA APLICACIÓN DE LOS LINEAMIENTOS GENERALES PARA EL EXPENDIO O DISTRIBUCIÓN DE ALIMENTOS Y BEBIDAS**

1. ¿Conoce Usted los Lineamientos generales para el expendio o distribución de alimentos y bebidas en los establecimientos de consumo escolar de los planteles de educación básica? (**No leer las opciones, esperar que responda y marcar la respuesta)**
2. Sí
3. No

Leer la siguiente oración de explicación:

**Los lineamientos son un conjunto de acciones de la SEP sobre la venta de alimentos y la realización de actividad física en la escuela.**

1. ¿Conoce Usted qué propició la creación de estos Lineamientos generales para el expendio o distribución de alimentos y bebidas? ? (**No leer las opciones, esperar que responda y marcar la o las opciones que se asemejen a su respuesta)**
2. Sobrepeso/obesidad
3. Diabetes
4. Sedentarismo
5. Falta de disponibilidad de agua simple potable
6. Amplia disponibilidad de alimentos con alto contenido calórico
7. No sabe
8. Otro (especificar)_____________________________________________________
9. ¿En qué consiste la aplicación de los Lineamientos generales para el expendio o distribución de alimentos y bebidas? (**No leer las opciones, esperar que responda y marcar la o las opciones que se asemejen a su respuesta)**
10. Regular la venta de alimentos y bebidas dentro de la escuela (o cualquier opción relacionada solo con alimentación y nutrición).
11. Mejorar la práctica de actividad en las escuelas (o cualquier opción relacionada sólo con activación física).
12. Promover un refrigerio saludable proporcionando información a los padres.
13. Privilegiar la disponibilidad y consumo de agua potable
14. No sabe
15. Otro (especificar)_____________________________________________________
16. ¿La aplicación de los Lineamientos generales para el expendio o distribución de alimentos y bebidas implicó algún cambio para su escuela? (**No leer las opciones, esperar que responda y marcar la respuesta)**
    1. Sí
    2. No **(pase a la pregunta 13)**
    3. No sabe **(pase a la pregunta 13)**
17. ¿Cuáles fueron los cambios? (**No leer las opciones, esperar que responda y marcar la o las opciones que se asemejen a su respuesta)**
18. Cambios relacionados con la venta de alimentos
19. Cambios relacionados con la venta de bebidas
20. Cambios relacionados con la disponibilidad de agua simple potable
21. Cambios relacionados con mayor activación física regular
22. Cambios relacionados con la promoción de una alimentación correcta
23. Cambios relacionados con la promoción de activación física regular
24. Cambios relacionados con el incremento de la participación del comité
25. Otro (especificar) _____________________________
26. ¿Podría mencionar en qué etapa de aplicación de los Lineamientos generales para el expendio o distribución de alimentos y bebidas nos encontramos en este ciclo escolar? (**No leer las opciones, esperar que responda y marcar la respuesta**)
27. Etapa I
28. Etapa II
29. Etapa III
30. No sabe
31. Otro (especificar)
32. ¿Conoce Usted los cambios que diferencian la etapa I de la II en los Lineamientos generales para el expendio o distribución de alimentos y bebidas? (**No leer las opciones, esperar que responda y marcar la respuesta**)
33. Sí
34. No **(pase a la pregunta 16)**
35. ¿Cuáles son? **(No leer las opciones, esperar que responda y marcar la o las opciones que se asemejen a su respuesta)**
    1. Disminución de **grasas, sales y azúcares** en los productos que se expenden en los establecimientos de consumo escolar
    2. Se **prioriza** la venta de **alimentos preparados**
    3. **Disminución de porciones** en los alimentos que se expenden
    4. **Eliminación** de la venta de algunos productos **industrializados**
    5. **Aumento** de consumo de **verduras y frutas**
    6. Otro. (Especificar)________________________________
36. Dentro de la escuela ¿alguna persona o personas supervisan la aplicación de los Lineamientos generales para el expendio o distribución de alimentos y bebidas? (**No leer las opciones, esperar que responda y marcar la respuesta**)
    1. Sí (**pase a la pregunta 17**)
    2. No (**pase a la pregunta 18**)
    3. No sabe (**pase a la pregunta 18**)
37. ¿Quién supervisa el cumplimiento? (**No leer las opciones, esperar que responda y marcar la o las opciones que se asemejen a su respuesta)**
38. Director
39. Profesores
40. Asociación de padres de familia
41. Supervisor de la Zona Escolar
42. Integrante(s) del Comité de Establecimientos del Consumo Escolar
43. Otro (especificar):
44. ¿Me podría mencionar las estrategias que Usted aplica para facilitar una alimentación correcta? (**No leer las opciones, esperar que responda y marcar la o las opciones que se asemejen a su respuesta)**
45. Difusión y promoción de la alimentación correcta en el aula, en la escuela y en el hogar
46. Sensibilización, capacitación y asesoría
47. Diseño de materiales de apoyo (carteles, trípticos, folletos, películas, etc.)
48. Diseño e implementación del programa de trabajo
49. Supervisión y seguimiento a la aplicación de las estrategias y acciones
50. Otras (especificar)

**NIVEL DE INFORMACION PARA LA APLICACIÓN DE LOS CRITERIOS NUTRIMENTALES**

1. ¿Podría mencionar cuáles son los principales criterios nutrimentales que definen los Lineamientos generales para el expendio o distribución de alimentos y bebidas? **(No leer las opciones, esperar que responda y marcar la o las opciones que se asemejen a su respuesta)**

| Criterio | Sí Menciona |
| --- | --- |
| Conformación de un refrigerio escolar que contribuya a una alimentación correcta para los alumnos. |  |
| Priorizar la venta de verduras y frutas. |  |
| Priorizar la venta de agua simple potable. |  |
| Priorizar la venta de alimentos preparados que favorecen una dieta correcta. |  |
| Que las características nutrimentales de los alimentos y bebidas sean de acuerdo a la cantidad de energía y nutrimentos que requieren los estudiantes para promover y mantener su salud. |  |
| Que las bebidas con edulcorantes no calóricos se vendan solo en secundarias *(en presentaciones de 250 ml sin cafeína y sin taurina).* |  |
| Que los alimentos cumplan con las normas higiénicas de preparación y consumo. |  |
| Que 1 vez por semana, se podrá sustituir el alimento preparado del refrigerio por alguna botana, pastelillo, postre, confite |  |
| Que 2 veces por semana, se podrá sustituir el alimento preparado del refrigerio por alimentos líquidos *(leche semidescremada, descremada, yogurt, alimentos líquidos de soya o jugos)*. |  |

1. ¿Podría mencionar qué alimentos debe incluir un refrigerio escolar saludable? **(No leer las opciones, esperar que responda y marcar la o las opciones que se asemejen a su respuesta)**

| Respuesta | Sí Menciona |
| --- | --- |
| Una o más porciones de verduras y frutas |  |
| Agua simple potable a libre demanda |  |
| Una porción de alimento preparado que cumplan con los criterios establecidos |  |
| Botanas dulces o saladas solo 1 vez a la semana. |  |
| Leche semidescremada o descremada, alimentos líquidos de soya, yogurt, jugos o néctares hasta 2 veces a la semana. |  |
| No sabe |  |
| Otra (especificar): ___________________________________________ |  |

**INFORMACIÓN SOBRE UTILIZACION DE LOS MATERIALES**

1. ¿Con qué materiales educativos cuenta Usted para aplicar los Lineamientos generales para el expendio o distribución de alimentos y bebidas y para el impulso de la activación física? (**No leer las opciones, esperar que responda y marcar la o las opciones que se asemejen a su respuesta)**
2. Manual para la preparación e higiene de alimentos y bebidas en los establecimientos de consumo escolar de los planteles de educación básica
3. Cómo preparar el refrigerio escolar y tener una alimentación correcta
4. Orientaciones para la regulación del expendio de alimentos y bebidas en las escuelas de educación básica.
5. Acuerdo mediante el cual se establecen los lineamientos generales para el expendio o distribución de alimentos y bebidas en los establecimientos de consumo escolar de los planteles de educación básica
6. Programa de Acción en el Contexto Escolar
7. Guías de activación física (guía primaria)
8. Guías de activación física (guía secundaria)
9. Otro (especificar)________________________________________
10. ¿Cuál de los siguientes materiales ha consultado? **(Mostrar las imágenes y marcar cuál o cuáles)**
11. |___|
12. |___|
13. |___|
14. |___|
15. |___|
16. |___|(Solamente para primaria)
17. |___| (Solamente para secundaria)

| **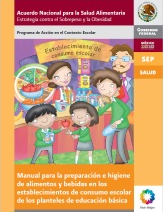**  **A** | 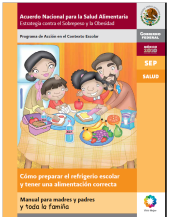  **B** | **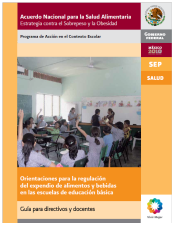**  **C** |
| --- | --- | --- |
| 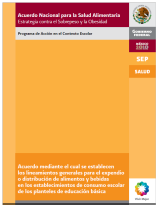  **D** | **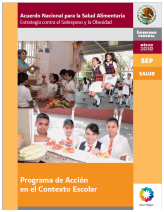**  **E** | **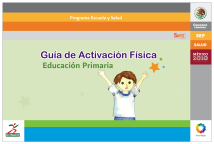**  **F** |
| 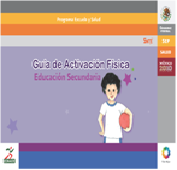  **G** |  |  |

1. ¿Qué tan útiles han sido los materiales para la implementación de los Lineamientos generales para el expendio o distribución de alimentos y bebidas? (**No leer las opciones, esperar que responda y marcar la opción que se asemeje a su respuesta)**
2. Muy útil
3. Útil
4. Poco útil (“más o menos”)
5. Nada útil
6. No sabe o no los conoce
7. No aplica (No los revisó)

**CONDICIONES QUE DIFICULTAN O FACILITAN LA APLICACIÓN DE LOS LINEAMIENTOS GENERALES DE PARA EL EXPENDIO O DISTRIBUCIÓN DE ALIMENTOS Y BEBIDAS EN LOS ESTABLECIMIENTOS DEL CONSUMO ESCOLAR**

1. ¿Qué tan de acuerdo está en que se ha logrado cumplir con las directivas de los Lineamientos generales para el expendio o distribución de alimentos y bebidas? (**No leer las opciones, esperar que responda y marcar la opción que se asemeje a su respuesta)**
2. Totalmente de acuerdo
3. De acuerdo
4. Ni de acuerdo ni en desacuerdo (indiferente)
5. En desacuerdo
6. Totalmente en desacuerdo
7. No sabe
8. Otro (especificar)____________________
9. De manera general, ¿Cuáles considera que son, en su escuela, los principales obstáculos o barreras para el cumplimiento de los Lineamientos generales para el expendio o distribución de alimentos y bebidas? **(No leer las opciones, esperar que responda y marcar la o las opciones que se asemeje a su respuesta)**
10. Desinformación o desconocimiento de los Lineamientos
11. Medidas poco adaptadas a la situación local
12. Poca supervisión a las escuelas por parte de las autoridades correspondientes
13. Convenios entre empresas y gobierno (poca credibilidad y contradicción)
14. Venta clandestina por parte de los niños o profesores
15. Presencia de puestos ambulantes afuera de escuela que no acatan los Lineamientos
16. Poco interés e involucramiento de padres
17. Preferencia de los niños hacia la comida chatarra
18. Resistencia al cambio de los profesores y/o directores
19. Hábitos/costumbres familiares poco saludables
20. Limitantes económicas
21. Influencia del marketing de alimentos y bebidas dirigida a los niños
22. Falta de apoyo por parte de las autoridades educativas
23. Falta de apoyo de las autoridades de salud
24. Falta de capacitación y asesoría
25. Falta de materiales de apoyo
26. Otro (especificar) ___________________________________________________
27. ¿Cuáles considera que son las condiciones que facilitan la implementación los Lineamientos generales para el expendio o distribución de alimentos y bebidas?  **(No leer las opciones, esperar que responda y marcar la opción que se asemeje a su respuesta)**
28. Entusiasmo y disposición de profesores
29. Responsabilidad/liderazgo de los directores
30. Reconocimiento de la comunidad escolar de ser una escuela transmisora de valores de auto cuidado y salud
31. Apoyo de los padres y madres de familia/ tutores
32. Apoyo por parte de los supervisores de la zona escolar
33. Participación activa de los comités
34. Capacitación y asesoría
35. Disponibilidad de materiales
36. Apoyo de autoridades educativas
37. Apoyo de autoridades de salud
38. Participación del Comité de establecimientos de consumo escolar
39. Otro (especificar)______

**INFORMACIÓN SOBRE EL FUNCIONAMIENTO Y ACTIVIDADES DEL COMITÉ DE IMPULSO A LA ACTIVACIÓN FÍSICA**

1. ¿Se realiza Activación Física dirigida en la escuela? **No leer las opciones, esperar que responda y marcar la respuesta)**
2. Sí
3. No **(pase a la pregunta 29)**
4. ¿En qué momento dentro del horario escolar se realiza la activación física?

| **Tipo de actividad** | **Número de días a la semana** | **Lugar en dónde la realizan** | **¿Quién la dirige?** | **¿Cuánto dura?** |
| --- | --- | --- | --- | --- |
| Activación al arrancar la jornada escolar |  |  |  |  |
| Activación dentro del salón de clase |  |  |  |  |
| Activación al final de la jornada |  |  |  |  |
| Recreo activo |  |  |  |  |
| Otro ( especificar) |  |  |  |  |

1. ¿Me podría mencionar las **estrategias** que utiliza para impulsar la activación física dentro de la escuela? **(esperar que responda y marcar la o las opciones que se asemejen a su respuesta)**
2. Difusión y promoción de la activación física, en el aula
3. Sensibilización, capacitación y asesoría
4. Diseño de materiales de apoyo (carteles, trípticos, folletos, películas etc.)
5. Diseño e implementación del programa de trabajo
6. Seguimiento y supervisión de la aplicación de las estrategias y acciones
7. Otro (especificar)

**CONDICIONES QUE DIFICULTAN O FACILITAN LA APLICACIÓN DE LOS LINEAMIENTOS EN EL IMPULSO DE LA ACTIVACION FISICA**

1. ¿Qué tan de acuerdo está en que se ha logrado cumplir con las directivas de los Lineamientos en su componente de activación física regular? (**No leer las opciones, esperar que responda y marcar la opción que se asemeje a su respuesta)**
2. Totalmente de acuerdo
3. De acuerdo
4. Ni de acuerdo ni en desacuerdo (indiferente)
5. En desacuerdo (más o menos)
6. Totalmente en desacuerdo
7. No sabe
8. Otro (especificar)____________________
9. De manera general, ¿Cuáles considera que son los principales obstáculos o barreras para la implementación de la activación física? (**No leer las opciones, esperar que responda y marcar la o las opciones que se asemejen a su respuesta)**
10. Desinformación o desconocimiento de los Lineamientos en cuanto a la activación física
11. Medidas poco adaptadas a la situación local
12. Poca supervisión a las escuelas por parte de las autoridades correspondientes
13. Falta de profesores de educación física
14. Resistencia de los profesores de grupo en llevar a cabo actividades físicas con sus alumnos
15. Poco interés e involucramiento de padres
16. Resistencia de los niños a hacer activación física regular
17. Resistencia al cambio de los profesores y/o directores
18. Falta de espacio dentro de la escuela
19. Falta de apoyo por parte de las autoridades educativas
20. Falta de apoyo de las autoridades de salud
21. Falta de capacitación y asesoría
22. Otro (especificar) ______

1. ¿Cuáles son los factores que facilitan la implementación de la activación física en su escuela? (**No leer las opciones, esperar que responda y marcar la o las opciones que se asemejen a su respuesta)**
2. Entusiasmo y disposición de profesores
3. Responsabilidad/liderazgo de los directores
4. Reconocimiento de la comunidad escolar de ser una escuela transmisora de valores de auto cuidado y salud
5. Apoyo de los padres y madres de familia/ tutores
6. Apoyo por parte de los supervisores de la zona escolar
7. Disponibilidad de materiales
8. Apoyo de autoridades educativas
9. Apoyo de autoridades de salud
10. Capacitación y asesoría
11. Participación activa de los comités
12. Otro (especificar) ______

**CARTILLA NACIONAL DE SALUD**

Finalmente, le haré dos preguntas relacionadas a la Cartilla Nacional de Salud

1. ¿Le entregaron las cartillas nacionales de salud para sus alumnos?
2. Sí
3. No
4. ¿Distribuyó las cartillas nacionales de salud a los padres/madres de familia o tutores?
5. Sí
6. No

**5 Questionnaire with school food vendors**

**INSTITUTO NACIONAL DE SALUD PÚBLICA**

**CENTRO DE INVESTIGACIÓN EN NUTRICIÓN Y SALUD**

**ESTUDIO SOBRE LA APLICACIÓN DE LA ETAPA II DE LOS LINEAMIENTOS GENERALES PARA EL EXPENDIO O DISTRIBUCIÓN DE ALIMENTOS Y BEBIDAS EN LOS ESTABLECIMIENTOS DE CONSUMO ESCOLAR DE LOS PLANTELES DE EDUCACIÓN BÁSICA**

**CUESTIONARIO PARA VENDEDORES Y EXPENDEDORES**

**FICHA DE IDENTIFICACIÓN**

1. Código del entrevistador |__|__| 2. Código de la escuela |__|__|

3. Clave de la escuela |__|__| 4. Localidad |__|__|

5. Municipio |__|__| 6. Estado |__|__|

7. Nombre de la escuela ____________________________________________

8. Turno: ______________________

9. Dirección de la escuela: ________________________________________________________________________________________________________________________________________________________________

10. Código de formato: |__|__|__|

11. Código del encuestado: |__|__|__|__|__|__|

1. Fecha de aplicación del cuestionario |__|__|__|__|__|__|

Día Mes Año

2. Nombre del vendedor o expendedor: ___________________________________________________________

**OBSERVACIÓN DEL ESTABLECIMIENTO / EXPENDIO**

1. Registra el tipo de establecimiento

*a. Expendio o tienda fija*

*b. Puesto ambulante*

*c. Cooperativa escolar*

*d. Cafetería*

1. Registra todos los tipos de alimentos que se expenden en el establecimiento:
2. Frutas y verduras
3. Alimentos preparados
4. Alimentos líquidos permitidos *(leche descremada, leche semidescremada, yogurt, lácteos fermentados, jugos, néctares, alimentos fermentados de soya y alimentos fermentados de soya con jugo)*
5. Leche entera
6. Bebidas para secundaria *(bebidas con edulcorantes no calóricos, sin cafeína ni taurina)*
7. Agua embotellada
8. Botanas dulces
9. Botanas saladas
10. Bebidas azucaradas

**INFORMACIÓN SOBRE UTILIZACIÓN DE LOS MATERIALES**

1.
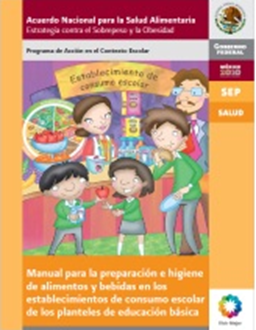
¿Conoce usted el *“Manual para la preparación e higiene de alimentos y bebidas en los establecimientos de consumo escolar de los planteles de educación básica”* ?

a. Sí

b. No **(pase a la pregunta 10)**

1. ¿Cuenta usted con un ejemplar del “Manual para la preparación e higiene de alimentos y bebidas en los establecimientos de consumo escolar de los planteles de educación básica”?
2. Sí
3. No **(pase a la pregunta 8)**

1. ¿Quién le proporcionó este manual? (**No leer las opciones, esperar que responda y marcar la opción que se asemeje a su respuesta)**
2. Comité de establecimiento de consumo escolar
3. Supervisor de zona escolar
4. Director de la escuela
5. Profesor (a) general
6. Profesor(a) encargada(o) de la cooperativa / de la venta escolar
7. Compañeros vendedores
8. Representante de alguna empresa de alimentos y/o bebidas
9. Lo consiguió por sí mismo(a)
10. Otro (especificar)
11. ¿Ha utilizado el “Manual para la preparación e higiene de alimentos y bebidas en los establecimientos de consumo escolar de los planteles de educación básica” durante el presente ciclo escolar?

a. Sí

b. No **(pase a la pregunta 10)**

1. ¿Qué tan útil han sido el “Manual para la preparación e higiene de alimentos y bebidas en los establecimientos de consumo escolar de los planteles de educación básica”?

a. Muy útil

b. Útil

c. Poco útil (más o menos)

d. Nada útil

e. No sabe o no los conoce

1. ¿Durante el presente ciclo escolar, ha utilizado la sección de menús para paquetes de refrigerios escolares que se incluye como anexo en el *“Manual para la preparación e higiene de alimentos y bebidas en los establecimientos de consumo escolar de los planteles de educación básica”* ?

a. Sí

b. No

1. ¿Cuenta usted con un listado de alimentos y bebidas que cumplen con los criterios nutrimentales de los lineamientos generales para vender en las escuelas?

a. Sí

b. No **(pase a la pregunta 13)**

En caso de SI, comprobar y especificar:

1. La mostró impresa
2. La mostró electrónica
3. Accede por internet (preguntar el link)
4. Otro (especificar) ____________________
5. No lo mostró al momento de la entrevista
6. ¿Quién le proporcionó este listado?
7. Consejo Escolar de Participación Social
8. Comité de Padres de Familia
9. Supervisor de zona escolar
10. Director de la escuela
11. Profesor(a) general
12. Profesor(a) encargada(o) de la cooperativa / de la venta escolar
13. Compañeros vendedores
14. Representante de alguna empresa de alimentos y/o bebidas
15. Lo consiguió por sí mismo(a)
16. Otro (especificar)
17. Además de los materiales de la Secretaría de Educación Pública, ¿Usted ha utilizado otros (manuales, videos, etc.) en relación con la alimentación?
18. Si
19. No **(pase a la pregunta 15)**
20. ¿Quién le proporcionó esos materiales?
    1. Gobiernos Estatales
    2. DIF
    3. Iniciativa privada
    4. Proveedores
    5. Prensa
    6. Otro (especificar)

**NIVEL DE INFORMACION PARA LA APLICACIÓN DE LOS CRITERIOS NUTRIMENTALES**

1. ¿Me podría mencionar cuáles son los principales criterios nutrimentales que definen los Lineamientos Generales para el expendio o distribución de alimentos y bebidas? **(No leer las opciones, esperar que responda y marcar la o las opciones que se asemejen a su respuesta)**

| Aspecto | Sí Menciona |
| --- | --- |
| Conformación de un refrigerio escolar que contribuya a una alimentación correcta para los alumnos. |  |
| Priorizar la venta de verduras y frutas. |  |
| Priorizar la venta de agua simple potable. |  |
| Priorizar la venta de alimentos preparados que facilitan una dieta correcta. |  |
| Que las características nutrimentales de los alimentos y bebidas sean de acuerdo a la cantidad de energía y nutrimentos que requieren los estudiantes para promover y mantener su salud. |  |
| Que las bebidas con edulcorantes no calóricos se vendan solo en secundarias. |  |
| Que los alimentos cumplan con las normas higiénicas de preparación y consumo. |  |
| Que 1 vez por semana, se podrán sustituir las preparaciones del refrigerio por alguna botana, pastelillo, confite o postre. |  |
| Que 2 veces por semana, se podrán sustituir las preparaciones del refrigerio por alimentos líquidos *(leche descremada, leche semidescremada, yogurt, lácteos fermentados, jugos, néctares y alimentos líquidos de soya)*. |  |

1. ¿Sabe en qué etapa de aplicación de los lineamientos escolares nos encontramos en este ciclo escolar? **(No leer respuestas. Marque solo una opción)**
2. Etapa 1
3. Etapa 2
4. Etapa 3
5. No sabe
6. Otra

1. ¿Conoce Usted los cambios llevados a cabo entre la etapa 1 y 2 de los lineamientos generales para el expendio o distribución de alimentos y bebidas?
2. Sí
3. No **(pase a la pregunta 19)**

18. ¿Cuáles son? **(No leer las opciones, esperar que responda y marcar la o las opciones que se asemejen a su respuesta)**

1. Disminución de calorías en las botanas dulces y saladas
2. Introducción de la limitación de las grasas saturadas en las botanas dulces y saladas
3. Introducción de la limitación del azúcar añadido en las botanas dulces y saladas
4. Introducción de la limitación del sodio añadido en las botanas saladas
5. Otro (especificar)
   - 1. ¿Sabe usted qué elementos debe incluir un refrigerio escolar saludable? **(No leer las opciones, esperar que responda y marcar la o las opciones que se asemejen a su respuesta)**

| Respuesta | Sí Menciona |
| --- | --- |
| Una o más porciones de frutas y verduras |  |
| Agua simple potable a libre demanda |  |
| Una porción de alimentos preparados que cumplan con los criterios establecidos |  |
| Botanas dulces o saladas solo 1 vez a la semana. |  |
| Leche descremada, semidescremada, yogurt, jugos o néctares, alimentos líquidos de soya hasta 2 veces a la semana. |  |
| No sabe |  |
| Otra (especificar): |  |

**PRÁCTICAS HIGIENICAS REFEREIDAS**

- - 1. Por favor, mencione las prácticas higiénicas que se deben seguir en los establecimientos de consumo escolar:

**(No leer las opciones, esperar que responda y marcar la o las opciones correspondientes)**

| Respuesta | Sí Menciona |
| --- | --- |
| Lavarse las manos con agua y jabón antes de preparar alimentos. |  |
| Llevar las uñas cortas y limpias |  |
| Cuidar la higiene de la vestimenta |  |
| Lavar y desinfectar frutas y verduras |  |
| Lavar y desinfectar utensilios de cocina |  |
| Usar cuchillos diferentes para alimentos crudos y cocidos. |  |
| Lavar todos los utensilios antes de preparar alimentos. |  |
| Lavar todos los utensilios después de preparar alimentos. |  |
| Que no haya animales domésticos en el establecimiento. |  |
| Que no haya plagas en el establecimiento. |  |
| Que el lugar donde se ofrecen los alimentos se encuentra alejado de fuentes de contaminación (por ejemplo basura, drenaje abierto, etc.). |  |
| Tapar la basura y eliminarla frecuentemente. |  |
| Que los recipientes y utensilios en donde se encuentran los alimentos estén limpios. |  |
| Mantener los alimentos tapados. |  |
| Refrigerar los alimentos que así lo requieran |  |
| Evitar toser, estornudar o picarse la nariz al preparar o estar en contacto con los alimentos. |  |
| No manejar de manera simultánea el dinero y los alimentos o utilizar guantes o una bolsa de plástico cuando se maneja dinero. |  |
| Cuidar la limpieza de los trapos de cocina. |  |
| Usar trapos de cocina diferentes por cada actividad. |  |
| Usar red para el cabello o gorra al preparar y manipular los alimentos. |  |
| Otra (especifique): |  |

**CONDICIONES QUE DIFICULTAN O FACILITAN LA APLICACIÓN DE LOS LINEAMIENTOS GENERALES PARA EL EXPENDIO O DISTRIBUCIÓN DE ALIMENTOS Y BEBIDAS EN LOS ESTABLECIMIENTOS DEL CONSUMO ESCOLAR**

21. De manera general, ¿Cuáles considera que son, en su escuela, los principales obstáculos o barreras para el cumplimiento de los lineamientos generales para el expendio o distribución de alimentos y bebidas? (**No leer las opciones, esperar que responda y marcar la o las opciones que se asemejen a su respuesta)**

1. Desinformación o desconocimiento de los Lineamientos
2. Medidas poco adaptadas a la situación local
3. Poca supervisión a las escuelas por parte de las autoridades correspondientes
4. Convenios entre empresas y gobierno (poca credibilidad y contradicción)
5. Venta clandestina por parte de los niños o profesores
6. Presencia de puestos ambulantes afuera de la escuela que no acatan los Lineamientos
7. Poco interés e involucramiento de padres
8. Preferencia de los niños hacia la comida chatarra
9. Resistencia al cambio de los profesores y/o directores
10. Hábitos/costumbres familiares poco saludables
11. Limitantes económicas
12. Influencia del marketing de alimentos y bebidas dirigida a los niños
13. Falta de apoyo por parte de las autoridades educativas
14. Falta de apoyo de las autoridades de salud
15. Falta de capacitación
16. Falta de materiales de apoyo
17. Otro (especificar) ___________________________________________________ _

22.¿Cuáles considera que son las condiciones que facilitan la implementación los lineamientos generales para el expendio o distribución de alimentos y bebidas**? (No leer las opciones, esperar que responda y marcar la o las opciones que se asemejen a su respuesta)**

1. Entusiasmo y disposición de profesores
2. Responsabilidad/liderazgo de los directores
3. Reconocimiento de la comunidad escolar de ser una escuela transmisora de valores de auto cuidado y salud
4. Apoyo de los padres y madres de familia/ tutores
5. Apoyo por parte de los supervisores de la zona escolar
6. Participación activa de los comités
7. Capacitación y asesoría
8. Disponibilidad de materiales
9. Apoyo de autoridades educativas
10. Apoyo de autoridades de salud
11. Participación del Comité de establecimientos de consumo escolar
12. Otro (especificar)______

**INFORMACIÓN SOBRE LA SUPERVISIÓN DE LA VENTA DE ALIMENTOS Y EL APOYO RECIBIDO PARA EL CUMPLIMIENTO DE LOS LINEAMIENTOS**

- - 1. ¿Sabe usted quién o quiénes son los encargados de supervisar el cumplimiento de los lineamientos generales para el expendio o distribución de alimentos y bebidas**? (Acepta más de una opción)**

1. Director
2. Profesores
3. Asociación de padres de familia
4. Supervisor de la Zona Escolar
5. Integrante(s) del Comité de Establecimientos del Consumo Escolar
6. Otro (especificar):

- - 1. ¿Su establecimiento de venta de alimentos y/o bebidas ha sido sujeto a alguna supervisión durante el presente ciclo escolar en relación al cumplimiento de los lineamientos escolares?

1. Si ¿Cuántas veces? ______
2. No **(pase a la pregunta 26)**
   - 1. ¿Qué rubros ha incluido dicha supervisión? **(Acepta más de una opción)**
3. Alimentos que cumplen y no cumplen con los criterios nutrimentales de los lineamientos generales
4. Calidad de los alimentos
5. Higiene personal
6. Higiene del establecimiento
7. Conocimientos sobre los lineamientos
8. Otra (especificar)
9. ¿Ha recibido usted capacitaciones sobre temas relacionados con la aplicación de los lineamientos durante el presente ciclo escolar?
10. Si
11. No  **(pase a la pregunta 28)**
12. ¿Sobre qué temas fueron esas capacitaciones? **(Acepta más de una opción)**
13. Lineamentos de la SEP en general (normatividad)
14. Nutrición y alimentación correcta
15. Sobrepeso y obesidad
16. Alimentos que cumplen y no cumplen con los criterios nutrimentales de los lineamientos generales
17. Higiene y manejo de alimentos
18. Promoción de la salud y actividad física
19. Promoción de algunos productos o marcas específicas de alimentos y/o bebidas
20. Otra (especificar)
21. ¿Ha recibido usted algún tipo de apoyo por parte del Comité del Establecimiento de Consumo Escolar, o su equivalente?
22. Si
23. No **(pase a la pregunta 30)**
24. ¿Qué tipo de apoyo ha recibido? **(Acepta más de una opción)**
25. Materiales didácticos
26. Orientación / capacitaciones / talleres / conferencias
27. Recursos materiales o económicos para mejorar la estructura física del establecimiento
28. Materias primas o productos para apoyar la venta de alimentos y/o bebidas
29. Apoyo para la promoción entre los alumnos de productos saludables
30. Otro (especificar)

**INFORMACIÓN SOBRE LA VENTA DE LOS ALIMENTOS DEL ESTABLECIMIENTO DE CONSUMO ESCOLAR**

1. ¿Usted ha realizado algún tipo de cambio en su establecimiento para cumplir con los lineamientos?
2. Si
3. No **(pase a la pregunta 32)**
4. Por favor mencione cuáles han sido esos cambios:

**(No leer las opciones, esperar que responda y marcar la o las opciones que se asemejen a su respuesta)**

1. Modificación de alimentos preparados para hacerlos más saludables y adecuados a la alimentación de los niños.
2. Incluir en la venta productos reconocidos como saludables *(frutas, verduras y agua simple)*
3. Eliminar la venta de productos industrializados que no cumplen con los criterios nutrimentales establecidos.
4. Eliminar la venta de bebidas azucaradas y/o refrescos.
5. Cambiar las presentaciones de productos industrializados por las nuevas versiones aprobadas que cumplen con los criterios nutrimentales de los lineamientos.
6. Limitar la venta de alimentos líquidos a solo 2 días a la semana (leche descremada y semidescremada, yogurt, jugos, néctares y alimentos líquidos de soya).
7. Limitar la venta de botanas, pastelillos, confites y postres a solo 1 día a la semana
8. Ofrecer paquetes armados que conformen un refrigerio escolar saludable.
9. Promover entre los alumnos el consumo de una alimentación saludable *(difusión, folletos, posters, etc.)*
10. Otra (especificar)
11. ¿Cuáles de los siguientes alimentos líquidos o bebidas ofrece en su establecimiento? ¿Cuántos días a la semana? ***(Preguntar por todos los incisos a continuación, a pesar de que no se observen a la venta en ese momento).***

a. Leche entera: No ( ) Sí ( ) ¿Cuántos días a la semana? ______

b. Leche descremada o semidescremada: No ( ) Sí ( ) ¿Cuántos días a la semana? ______

c. Yogurt: No ( ) Sí ( ) ¿Cuántos días a la semana? ______

d. Jugos de fruta: No ( ) Sí ( ) ¿Cuántos días a la semana? ______

e. Néctares: No ( ) Sí ( ) ¿Cuántos días a la semana? ______

f. Jugos de verduras: No ( ) Sí ( ) ¿Cuántos días a la semana? ______

g. Alimentos líquidos de soya: No ( ) Sí ( ) ¿Cuántos días a la semana? ______

h. Alimento lácteo fermentado: No ( ) Sí ( ) ¿Cuántos días a la semana? _____

i. Bebidas azucaradas: No ( ) Sí ( ) ¿Cuántos días a la semana? ______

j. Refrescos o bebidas específicas para alumnos de secundaria (bebidas con edulcorantes no calóricos, sin cafeína ni taurina): No ( ) Sí ( ) ¿Cuántos días a la semana? ______

1. ¿Cuáles de los siguientes alimentos ofrece en su establecimiento? ¿Cuántos días a la semana? ***(Preguntar por todos los incisos a continuación, a pesar de que no se observen a la venta en ese momento)***

a. Verduras y frutas: No ( ) Sí ( ) ¿Cuántos días a la semana? ______

b. Alimentos preparados no fritos: No ( ) Sí ( ) ¿Cuántos días a la semana? ______

c. Alimentos preparados fritos: No ( ) Sí ( ) ¿Cuántos días a la semana? ______

d. Alimentos industrializados no fritos (botanas no fritas): No ( ) Sí ( ) ¿Cuántos días a la semana? ______

e. Alimentos industrializados fritos (botanas fritas): No ( ) Sí ( ) ¿Cuántos días a la semana? ______

f. Dulces, pastelillos, chicles o confites: No ( ) Sí ( ) ¿Cuántos días a la semana? ______

1. ¿Cuáles son productos que más compran los alumnos en su establecimiento de consumo escolar? **(Acepta solo una opción)*.***

a. Verduras y frutas ( )

b. Alimentos preparados no fritos ( )

c. Alimentos preparados fritos ( )

d. Alimentos industrializados no fritos *(botanas no fritas)* ( )

e. Alimentos industrializados fritos *(botanas fritas)* ( )

f. Dulces, pastelillos, chicles o confites ( )

g. Alimentos líquidos (Leche, jugo, néctar, yogurt, líquidos de soya) ( )

h. Agua simple potable ( )

i. Bebidas con edulcorantes no calóricos ( )

j. Otras bebidas ( )

**Observaciones:**

________________________________________________________________________________________________________________________________________________________________

**6 Questionnaire with Principal**

**INSTITUTO NACIONAL DE SALUD PÚBLICA**

**CENTRO DE INVESTIGACIÓN EN NUTRICIÓN Y SALUD**

**ESTUDIO SOBRE LA APLICACIÓN DE LA ETAPA II DE LOS LINEAMIENTOS GENERALES PARA EL EXPENDIO O DISTRIBUCIÓN DE ALIMENTOS Y BEBIDAS EN LOS ESTABLECIMIENTOS DE CONSUMO ESCOLAR DE LOS PLANTELES DE EDUCACIÓN BÁSICA**

**CUESTIONARIO PARA DIRECTORES**

**FICHA DE IDENTIFICACIÓN**

1. Código del entrevistador |__|__| 2. Código de la escuela |__|__|

3. Clave de la escuela |__|__| 4. Localidad |__|__|

5. Municipio |__|__| 6. Estado |__|__|

7. Nombre de la escuela: __________________________________________________________

8. Turno: ______________________

9. Dirección de la escuela: ________________________________________________________________________________________________________________________________________________________________

10. Código del formato: |__|__|__|

11. Código del encuestado: |__|__|__|__|__|__|

12. Fecha de aplicación del cuestionario |__|__|__|__|__|__|

Día Mes Año

13. Nombre del director: ________________________________________________

14. Número de alumnos en la escuela: _______________________

**INFORMACIÓN RELATIVA A LA APLICACIÓN DE LA ETAPA II DE LOS LINEAMIENTOS GENERALES PARA EL EXPENDIO O DISTRIBUCIÓN DE ALIMENTOS Y BEBIDAS EN LOS ESTABLECIMIENTOS DE CONSUMO ESCOLAR DE LOS PLANTELES DE EDUCACIÓN BÁSICA**

1. ¿Conoce usted los Lineamientos generales para el expendio o distribución de alimentos y bebidas en los establecimientos de consumo escolar de los planteles de educación básica? (**No leer las opciones, esperar que responda y marcar la respuesta)**
   1. Sí
   2. No

**Leer la siguiente oración de explicación:**

**Los lineamientos son un conjunto de acciones de la SEP sobre la venta de alimentos y la realización de actividad física en la escuela.**

1. ¿Conoce usted qué propició la creación de estos Lineamientos generales para el expendio o distribución de alimentos y bebidas? (**No leer las opciones, esperar que responda y marcar la o las opciones que se asemejen a su respuesta)**
2. Sobrepeso/obesidad
3. Diabetes
4. Sedentarismo
5. Falta de disponibilidad de agua simple potable
6. Amplia disponibilidad de alimentos con alto contenido calórico
7. No sabe
8. Otro (especificar)___________________________________________________
9. ¿En qué consiste la aplicación de los Lineamientos generales para el expendio o distribución de alimentos y bebidas? (**No leer las opciones, esperar que responda y marcar la o las opciones que se asemejen a su respuesta)**
10. Regular la venta de alimentos y bebidas dentro de la escuela (o cualquier opción relacionada sólo con alimentación y nutrición).
11. Mejorar la práctica de actividad en las escuelas (o cualquier opción relacionada sólo con activación física).
12. Promover un refrigerio saludable proporcionando información a los padres.
13. Privilegiar la disponibilidad y consumo de agua potable
14. No sabe
15. Otro (especificar)_____________________________________________________
16. ¿La aplicación de los Lineamientos generales para el expendio o distribución de alimentos y bebidas implicó algún cambio para su escuela? (**No leer las opciones, esperar que responda y marcar la respuesta)**
    1. Sí
    2. No **(pase a la pregunta 6)**
    3. No sabe **(pase a la pregunta 6)**
17. ¿Cuáles fueron los cambios? **(No leer las opciones, esperar que responda y marcar la o las opciones que se asemejen a su respuesta)**
18. Cambios relacionados con la venta de alimentos
19. Cambios relacionados con la venta de bebidas
20. Cambios relacionados con la disponibilidad de agua simple potable
21. Cambios relacionados con la activación física regular
22. Cambios relacionados con la promoción de una alimentación correcta
23. Cambios relacionados con la promoción de activación física regular
24. Cambios relacionados con el incremento de la participación del comité
25. Otro (especificar) _____________________________
26. ¿Podría mencionar en qué etapa de aplicación de los Lineamientos generales para el expendio o distribución de alimentos y bebidas nos encontramos en este ciclo escolar? (**No leer las opciones, esperar que responda y marcar la respuesta**)
27. Etapa I
28. Etapa II
29. Etapa III
30. No sabe
31. Otro (especificar)
32. ¿Conoce usted los cambios que diferencian la etapa I de la II de los Lineamientos generales para el expendio o distribución de alimentos y bebidas? (**No leer las opciones, esperar que responda y marcar la respuesta)**
33. Sí
34. No **(pase a la pregunta 9)**
35. ¿Cuáles son? **(No leer las opciones, esperar que responda y marcar la o las opciones que se asemejen a su respuesta)**
    1. Disminución **de grasas, sales y azúcares** en los productos que se expenden en los establecimientos de consumo escolar
    2. Se **prioriza** la venta de **alimentos preparados**
    3. **Disminución de porciones** en los alimentos que se expenden
    4. **Eliminación** de la venta de algunos **productos industrializados**
    5. **Aumento** de consumo de **verduras y frutas**
    6. Otro. (Especificar)________________________________
36. Dentro de la escuela ¿alguna persona o personas supervisan la aplicación de los Lineamientos generales para el expendio o distribución de alimentos y bebidas? (**No leer las opciones, esperar que responda y marcar la respuesta)**
    1. Sí
    2. No (**pase a la pregunta 11**)
    3. No sabe (**pase a la pregunta 11**)
37. ¿Quién supervisa el cumplimiento? (**No leer las opciones, esperar que responda y marcar la o las opciones que se asemejen a su respuesta)**
38. Director
39. Profesores
40. Asociación de padres de familia
41. Supervisor de la Zona Escolar
42. Integrante(s) del Comité de Establecimientos del Consumo Escolar
43. Otro (especificar):
44. ¿Fuera o enfrente de la escuela hay comercio ambulante de alimentos y bebidas? (**No leer las opciones, esperar que responda y marcar la respuesta)**
45. Sí
46. No

**NIVEL DE INFORMACION PARA LA APLICACIÓN DE LOS CRITERIOS NUTRIMENTALES**

1. ¿Podría mencionar cuáles son los principales criterios nutrimentales que definen los Lineamientos generales para el expendio o distribución de alimentos y bebidas? **(No leer las opciones, esperar que responda y marcar la o las opciones que se asemejen a su respuesta)**

| Criterio | Sí Menciona |
| --- | --- |
| Conformación de un refrigerio escolar que contribuya a una alimentación correcta para los alumnos. |  |
| Priorizar la venta de verduras y frutas. |  |
| Priorizar la venta de agua simple potable. |  |
| Priorizar la venta de alimentos preparados que favorecen una dieta correcta. |  |
| Que las características nutrimentales de los alimentos y bebidas sean de acuerdo a la cantidad de energía y nutrimentos que requieren los estudiantes para promover y mantener su salud. |  |
| Que las bebidas con edulcorantes no calóricos se vendan solo en secundarias *(en presentaciones de 250 ml y sin cafeína).* |  |
| Que los alimentos cumplan con las normas higiénicas de preparación y consumo. |  |
| Que 1 vez por semana, se podrá sustituir el alimento preparado del refrigerio por alguna botana, pastelillo, postre, confite. |  |
| Que 2 veces por semana, se podrá sustituir el alimento preparado del refrigerio por alimentos líquidos *(leche semidescremada, descremada, yogurt, alimentos líquidos de soya o jugos)*. |  |

1. ¿Podría mencionar qué alimentos debe incluir un refrigerio escolar saludable? **(No leer las opciones, esperar que responda y marcar la o las opciones que se asemejen a su respuesta)**

| Respuesta | Sí Menciona |
| --- | --- |
| Una o más porciones de verduras y frutas. |  |
| Agua simple potable a libre demanda. |  |
| Una porción de alimento preparado que cumpla con los criterios establecidos. |  |
| Botanas dulces o saladas solo 1 vez a la semana. |  |
| Leche semidescremada o descremada, alimentos líquidos de soya, yogurt, jugos o néctares hasta 2 veces a la semana. |  |
| No sabe. |  |
| Otra (especificar): ___________________________________________ |  |

1. ¿Hay alimentos que NO se venden todos los días? (**No leer las opciones, esperar que responda y marcar la respuesta)**
2. Sí
3. No (**pase a la pregunta 16**)
4. No sabe (**pase a la pregunta 16**)
5. ¿Qué alimentos son los que NO se venden todos los días? **(No leer las opciones, esperar que responda y marcar la o las opciones que se asemejen a su respuesta)**
6. Verduras y frutas ¿cuántos días NO se venden? _____
7. Alimentos preparados ¿cuántos días NO se venden? _____
8. Alimentos líquidos permitidos *(leche descremada, leche semidescremada, yogurt, lácteos fermentados, de soya, jugos y néctares)*  ¿cuántos días NO se venden? _____
9. Leche entera ¿cuántos días NO se venden? _____
10. Bebidas para secundaria ¿cuántos días NO se venden? _____
11. Agua embotellada ¿cuántos días NO se venden? _____
12. Botanas dulces ¿cuántos días NO se venden? _____
13. Botanas saladas ¿cuántos días NO se venden? _____
14. Otros (especificar nombre del producto) __ ¿cuántos días NO se venden? ________
15. ¿En este año, cree usted que la calidad de los alimentos y bebidas que se venden en esta escuela? (**Leer las opciones**)
16. Ha mejorado
17. Sigue igual
18. Ha empeorado
19. No sabe
20. ¿Qué medidas se implementan para asegurar la disponibilidad permanente del agua simple potable para el consumo de los alumnos? **(No leer las opciones, esperar que responda y marcar la o las opciones que se asemejen a su respuesta)**
21. Gestión con las autoridades municipales, entre otros, para crear condiciones que aseguren la disponibilidad de agua simple potable
22. Gestiones a través del Consejo Escolar de Participación Social (CEPS)
23. Colaboración de padres de familia
24. Contrato con proveedores
25. Otro (especificar)________________________________________

**INFORMACIÓN SOBRE LA UTILIZACIÓN DE LOS MATERIALES EDUCATIVOS**

1. ¿Con qué materiales educativos cuenta la escuela para aplicar los Lineamientos generales para el expendio o distribución de alimentos y bebidas y para el impulso de la activación física? (**No leer las opciones, esperar que responda y marcar la o las opciones que se asemejen a su respuesta)**
2. Manual para la preparación e higiene de alimentos y bebidas en los establecimientos de consumo escolar de los planteles de educación básica
3. Cómo preparar el refrigerio escolar y tener una alimentación correcta
4. Orientaciones para la regulación del expendio de alimentos y bebidas en las escuelas de educación básica (guía para directivos y docentes)
5. Acuerdo mediante el cual se establecen los Lineamientos generales para el expendio o distribución de alimentos y bebidas en los establecimientos de consumo escolar de los planteles de educación básica
6. Programa de Acción en el Contexto Escolar
7. Guías de activación física (guía primaria y secundaria)
8. Ninguno de los anteriores
9. Otro (especificar)________________________________________
10. ¿Cuál de los siguientes materiales ha consultado? **(Mostrar las imágenes y marcar cuál o cuáles)**
11. |___|
12. |___|
13. |___|
14. |___|
15. |___|
16. |___| (Solamente para primaria)
17. |___| (Solamente para secundaria**)**

| **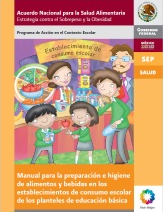**  **A** | 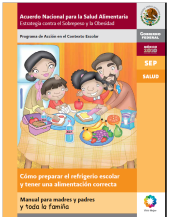  **B** | **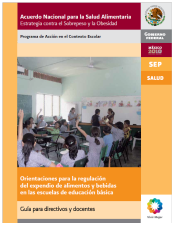**  **C** |
| --- | --- | --- |
| 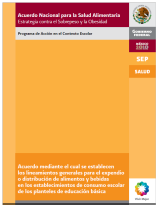  **D** | **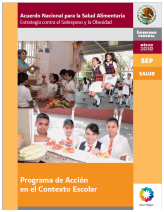**  **E** | **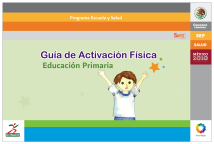**  **F** |
| 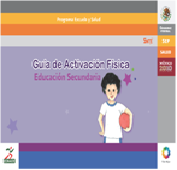  **G** |  |  |

1. ¿Qué tan útiles han sido los materiales para la implementación de los Lineamientos generales para el expendio o distribución de alimentos y bebidas? (**No leer las opciones, esperar que responda y marcar la opción que se asemeje a su respuesta)**
2. Muy útil
3. Útil
4. Poco útil (“más o menos”)
5. Nada útil
6. No sabe o no los conoce
7. No aplica (No los revisó)
8. ¿A quién o a quiénes los distribuyó?

|  | Profesor | Padres de familia | Responsables del comité de establecimientos de consumo escolar | Responsables del comité de impulso a la activación física | Responsable del establecimiento de consumo escolar | Otro  (especificar) |
| --- | --- | --- | --- | --- | --- | --- |
| Manual para la preparación e higiene de alimentos y bebidas en los establecimientos de consumo escolar de los planteles de educación básica |  |  |  |  |  |  |
| Cómo preparar el refrigerio escolar y tener una alimentación correcta |  |  |  |  |  |  |
| Orientaciones para la regulación del expendio de alimentos y bebidas en las escuelas de educación básica |  |  |  |  |  |  |
| Acuerdo mediante el cual se establecen los Lineamientos generales para el expendio o distribución de alimentos y bebidas en los establecimientos de consumo escolar de los planteles de educación básica |  |  |  |  |  |  |
| Programa de Acción en el Contexto Escolar |  |  |  |  |  |  |
| Guía de Activación Física |  |  |  |  |  |  |

1. ¿Conoce la página WEB que contiene la guía de alimentos y bebidas escolares, que cumplen con los lineamientos? (**No leer las opciones, esperar que responda y marcar la respuesta)**
2. Sí
3. No (**pase a la pregunta 24**)
4. ¿Qué información ha consultado en la página? **(No leer las opciones, esperar que responda y marcar la o las opciones que se asemejen a su respuesta)**
5. Criterios nutrimentales
6. Guía de alimentos y bebidas que cumplen con los criterios nutrimentales
7. Alimentación correcta
8. Preparación de alimentos
9. Otro. Especificar ________________________________

**INFORMACIÓN SOBRE EL FUNCIONAMIENTO Y ACTIVIDADES DEL CONSEJO ESCOLAR DE PARTICIPACIÓN SOCIAL (CEPS)**

1. ¿En la escuela, existe el Consejo Escolar de Participación Social? (**No leer las opciones, esperar que responda y marcar la respuesta)**
2. Sí (**pase a la pregunta 26**)
3. No (**pase a la pregunta 25**)
4. No sabe (**pase a la pregunta 25**)
5. En caso de no existir CEPS, ¿qué figura existe?
6. Asociación de padres de familia
7. Otro (especificar)________

**Pase a la pregunta 28**

1. ¿Quiénes lo conforman? **(acepta más de una opción)**

|  | Se identificó a: | ¿cuántos son? |
| --- | --- | --- |
| Director |  |  |
| Padres y madres y tutores de familia |  |  |
| Personal de apoyo y asistencia a la educación |  |  |
| Profesores |  |  |
| Ex-alumnos |  |  |
| Otro (especificar) ___________________ |  |  |

1. ¿Cuántas veces se ha reunido el CEPS durante este ciclo escolar? (**No leer las opciones, esperar que responda y marcar la opción que se asemejen a su respuesta)**
2. No se ha reunido
3. De 1-3 veces
4. 4 a 5 veces
5. 6 veces o más
6. No sabe

**INFORMACIÓN SOBRE EL FUNCIONAMIENTO Y ACTIVIDADES DEL COMITÉ DE ESTABLECIMIENTOS DE CONSUMO ESCOLAR**

1. ¿En esta escuela existe un Comité de Establecimientos de Consumo Escolar? (**No leer las opciones, esperar que responda y marcar la respuesta)**
2. Sí
3. No **(pase a la pregunta 32)**
4. ¿Quiénes conforman el Comité de Establecimientos de Consumo Escolar?

|  | Se identificó a: | ¿cuántos son? |
| --- | --- | --- |
| Director |  |  |
| Padres y madres o tutores de familia |  |  |
| Personal de apoyo y asistente de educación |  |  |
| Profesores |  |  |
| Alumnos |  |  |
| Otro (especificar) ___________________ |  |  |

1. ¿Cuáles son las funciones del Comité de establecimientos de consumo escolar? (**No leer las opciones, esperar que responda y marcar la o las opciones que se asemejen a su respuesta**)
2. Contribuir en la promoción de entornos seguros y saludables mediante la implementación de prácticas de higiene en el establecimiento escolar.
3. Supervisar que el establecimiento de consumo escolar ofrezca a los alumnos refrigerios escolares que cumplan con los criterios nutrimentales establecidos en los Lineamientos generales para el expendio o distribución de alimentos y bebidas de los establecimientos de consumo escolar.
4. Coordinarse con el personal de la escuela para promover  una nueva cultura para el cuidado de la salud de los escolares.
5. Supervisar la aplicación sistemática de los Lineamientos generales para el expendio o distribución de los alimentos y bebidas en los establecimientos de consumo escolar.
6. Supervisar y vigilar permanentemente la calidad de los alimentos y bebidas que se expenden en los establecimientos de consumo escolar (higiene, costo, orden y seguridad).
7. Propiciar la colaboración de los padres de familia o tutores y sus asociaciones con el resto de la comunidad educativa, del sector salud para el desarrollo de acciones de capacitación a la comunidad escolar para el favorecimiento de una alimentación correcta dentro y fuera de la escuela.
8. Promover el consumo de agua simple potable
9. No sabe
10. Otro (especificar): ____________
11. ¿Cuántas veces se ha reunido el Comité de Establecimientos de Consumo Escolar durante este periodo escolar? (**No leer las opciones, esperar que responda y marcar la opción que se asemeje a su respuesta)**
12. No se ha reunido
13. De 1 a 3 veces
14. 4 a 5 veces
15. 6 veces o más
16. No sabe
17. No aplica
18. ¿Quién o quiénes verifican los alimentos que se venden en la escuela? **(No leer las opciones, esperar que responda y marcar la o las opciones que se asemejen a su respuesta)**
19. Director
20. Profesores
21. Responsable del establecimiento de consumo escolar
22. Padres de familia
23. Integrantes del Comité de establecimientos de consumo escolar
24. Nadie
25. Otro (especificar) ____________________
26. ¿Cuenta usted con el listado de alimentos y bebidas que cumplen con los criterios nutrimentales de los Lineamientos? (**No leer las opciones, esperar que responda y marcar la respuesta)**
27. Sí
28. No (**pase a la pregunta 35**)
29. En caso de SI, comprobar y especificar:
30. La mostró impresa
31. La mostró electrónica
32. Accede por internet (preguntar el link)
33. Otro (especificar) ____________________

**Pase a la pregunta 36**

1. En caso de NO comprobar que cuenta con el listado, explorar la razón:
2. No sabe a qué lista se refieren
3. No la tienen
4. La conocen pero no la usan
5. Otro (especificar) _________________________________________
6. ¿Me podría mencionar las estrategias que se aplican en la escuela para facilitar una alimentación correcta? (**No leer las opciones, esperar que responda y marcar la o las opciones que se asemejen a su respuesta**)
   1. Difusión y promoción de la alimentación correcta en el aula, en la escuela y en el hogar
   2. Sensibilización, capacitación y asesoría
   3. Diseño de materiales de apoyo (carteles, trípticos, folletos, películas, etc.)
   4. Diseño e implementación del programa de trabajo
   5. Supervisión y seguimiento a la aplicación de las estrategias y acciones
   6. Otras (especificar)
7. Para la implementación de los Lineamientos generales, ¿qué actores de la comunidad escolar participan en cada una de las estrategias siguientes:? (leer la estrategia y **preguntar por cada actor**)

|  | Difusión y promoción de la alimentación correcta en el aula, en la escuela y en el hogar | Sensibilización, capacitación y asesoría | Diseño de materiales de apoyo (carteles, trípticos, folletos, etc.) | Diseño e implementación del programa de trabajo | Supervisión y seguimiento a la aplicación de las estrategias y acciones | Otras (especificar) |
| --- | --- | --- | --- | --- | --- | --- |
| Profesores |  |  |  |  |  |  |
| Responsables del establecimiento escolar |  |  |  |  |  |  |
| Padres de familia |  |  |  |  |  |  |

**CONDICIONES QUE DIFICULTAN O FACILITAN LA APLICACIÓN DE LOS LINEAMIENTOS GENERALES DE PARA EL EXPENDIO O DISTRIBUCIÓN DE ALIMENTOS Y BEBIDAS EN LOS ESTABLECIMIENTOS DE CONSUMO ESCOLAR**

1. ¿Qué tan de acuerdo está en que se ha logrado cumplir con las directivas de los Lineamientos generales para el expendio o distribución de alimentos y bebidas? **(No leer las opciones, esperar que responda y marcar la opción que se asemeje a su respuesta)**
2. Totalmente de acuerdo
3. De acuerdo
4. Ni de acuerdo ni en desacuerdo (indiferente)
5. En desacuerdo
6. Totalmente en desacuerdo
7. No sabe
8. Otro (especificar)____________________
9. De manera general, ¿Cuáles considera que son, en su escuela, los principales obstáculos o barreras para el cumplimiento de los Lineamientos generales para el expendio o distribución de alimentos y bebidas? **(No leer las opciones, esperar que responda y marcar la o las opciones que se asemejen a su respuesta)**
10. Desinformación o desconocimiento de los Lineamientos
11. Medidas poco adaptadas a la situación local
12. Poca supervisión a las escuelas por parte de las autoridades correspondientes
13. Convenios entre empresas y gobierno (poca credibilidad y contradicción)
14. Venta clandestina por parte de los niños o profesores
15. Presencia de puestos ambulantes afuera de la escuela que no acatan los Lineamientos
16. Poco interés e involucramiento de padres
17. Preferencia de los niños hacia la comida chatarra
18. Resistencia al cambio de los profesores y/o directores
19. Hábitos/costumbres familiares poco saludables
20. Limitantes económicas
21. Influencia del marketing de alimentos y bebidas dirigida a los niños
22. Falta de apoyo por parte de las autoridades educativas
23. Falta de apoyo de las autoridades de salud
24. Falta de capacitación
25. Falta de materiales de apoyo
26. Otro (especificar) ___________________________________________________ _
27. ¿Cuáles considera que son las condiciones que facilitan la implementación de los Lineamientos generales para el expendio o distribución de alimentos y bebidas?  **(No leer las opciones, esperar que responda y marcar la o las opciones que se asemejen a su respuesta)**
28. Entusiasmo y disposición de profesores
29. Responsabilidad/liderazgo de los directores
30. Reconocimiento de la comunidad escolar de ser una escuela transmisora de valores de auto cuidado y salud
31. Apoyo de los padres y madres de familia/ tutores
32. Apoyo por parte de los supervisores de la zona escolar
33. Participación activa de los comités
34. Capacitación y asesoría
35. Disponibilidad de materiales
36. Apoyo de autoridades educativas
37. Apoyo de autoridades de salud
38. Participación del Comité de establecimientos de consumo escolar
39. Otro (especificar)______

**INFORMACIÓN SOBRE EL FUNCIONAMIENTO Y ACTIVIDADES DEL COMITÉ DE IMPULSO A LA ACTIVACIÓN FÍSICA**

1. En esta escuela ¿Hay Comité de Impulso a la Activación Física? (**No leer las opciones, esperar que responda y marcar la respuesta)**
2. Sí
3. No **(pase a la pregunta 44)**
4. ¿Quiénes conforman el Comité de Impulso a la Activación Física?

|  | Se identifico a: | ¿cuántos son? |
| --- | --- | --- |
| Director |  |  |
| Padres y madres o tutores de familia |  |  |
| Personal de apoyo y asistente de educación |  |  |
| Profesores |  |  |
| Alumnos |  |  |
| Otro (especificar) ___________________ |  |  |

1. ¿Cuántas veces se ha reunido el Comité de Impulso a la Activación Física durante este ciclo escolar? **(No leer las opciones, esperar que responda y marcar la opción que se asemeje a su respuesta)**
2. No se ha reunido
3. De 1 a 3 veces
4. 4 a 5 veces
5. 6 veces o más
6. No sabe
7. ¿Cuáles son las funciones del Comité de Impulso a la activación física? **(No leer las opciones, esperar que responda y marcar la o las opciones que se asemejen a su respuesta)**
8. Incentivar la implementación y uso de las Guías de Activación Física para profesores de educación básica
9. Promover la recuperación de espacios seguros en la escuela y en lugares cercanos a ella, para el impulso de la activación física regular
10. Buscar recursos de apoyo, personal especializado e insumos básicos para la implementación de la activación física regular en la escuela
11. Gestionar acciones y apoyos con instancias externas a la escuela para favorecer la activación física
12. Otro (especificar): ________________
13. ¿Se realiza Activación Física regular en la escuela? (**No leer las opciones, esperar que responda y marcar la respuesta)**
14. Sí
15. No
16. ¿En qué momento dentro del horario escolar se realiza la activación física?

| **Tipo de actividad** | **Número de días a la semana** | **Lugar en dónde la realizan** | **¿Quién la dirige?** | **¿Cuánto dura?** |
| --- | --- | --- | --- | --- |
| Activación al arrancar la jornada escolar |  |  |  |  |
| Activación dentro del salón de clase |  |  |  |  |
| Activación al final de la jornada |  |  |  |  |
| Recreo activo |  |  |  |  |
| Otro ( especificar) |  |  |  |  |

1. ¿Me podría mencionar las estrategias que utiliza para impulsar la activación física dentro de la escuela? **(No leer las opciones, esperar que responda y marcar la o las opciones que se asemejen a su respuesta**)
2. Difusión y promoción de la activación física, en el aula, en la escuela y en el hogar
3. Sensibilización, capacitación y asesoría
4. Diseño de materiales de apoyo (carteles, trípticos, folletos, películas, etc.)
5. Diseño e implementación del programa de trabajo
6. Seguimiento y evaluación a la aplicación de las estrategias y acciones
7. Otras (especificar)

**CONDICIONES QUE DIFICULTAN O FACILITAN LA APLICACIÓN DE LOS LINEAMIENTOS EN EL IMPULSO DE LA ACTIVACION FISICA**

1. ¿Qué tan de acuerdo está en qué se ha logrado cumplir con las directivas de los Lineamientos en su componente de activación física regular? (**No leer las opciones, esperar que responda y marcar la opción que se asemeje a su respuesta)**
2. Totalmente de acuerdo
3. De acuerdo
4. En desacuerdo
5. Ni de acuerdo ni en desacuerdo (indiferente)
6. Totalmente en desacuerdo
7. No sabe
8. Otro (especificar)____________________
9. ¿Cuáles considera que son los principales obstáculos o barreras para la implementación de la activación física? **(No leer las opciones, esperar que responda y marcar la o las opciones que se asemejen a su respuesta)**
10. Desinformación o desconocimiento de los Lineamientos en cuanto a la activación física
11. Medidas poco adaptadas a la situación local
12. Poca supervisión a las escuelas por parte de las autoridades correspondientes
13. Falta de profesores de educación física
14. Resistencia de los profesores de grupo en llevar a cabo actividades físicas con sus alumnos
15. Poco interés e involucramiento de padres
16. Resistencia de los niños a hacer activación física regular
17. Resistencia al cambio de los profesores y/o directores
18. Falta de espacio dentro de la escuela
19. Falta de apoyo por parte de las autoridades educativas
20. Falta de apoyo de las autoridades de salud
21. Falta de capacitación y asesoría
22. Otro (especificar) ______
23. ¿Cuáles son los factores que facilitan la implementación de la activación física? **(No leer las opciones, esperar que responda y marcar la o las opciones que se asemejen a su respuesta)**
24. Entusiasmo y disposición de profesores
25. Responsabilidad/liderazgo de los directores
26. Reconocimiento de la comunidad escolar de ser una escuela transmisora de valores de auto cuidado y salud
27. Apoyo de los padres y madres de familia/ tutores
28. Apoyo por parte de los supervisores de la zona escolar
29. Disponibilidad de materiales
30. Apoyo de autoridades educativas
31. Apoyo de autoridades de salud
32. Capacitación y asesoría
33. Participación activa de los comités
34. Otro (especificar) ______

**PROMOCIÓN DE LA SALUD PARA LA COMUNIDAD ESCOLAR**

1. En el último año ¿La comunidad escolar de esta escuela ha recibido alguna capacitación sobre el cuidado de la salud? (**No leer las opciones, esperar que responda y marcar la respuesta)**
2. Sí
3. No **(pase a la pregunta 54)**
4. ¿Sobre qué temáticas? (**No leer las opciones, esperar que responda y marcar la o las opciones que se asemejen a su respuesta)**
5. Lineamientos generales
6. Alimentación correcta
7. Activación física
8. Higiene y saneamiento básico
9. Cuidado del ambiente
10. Convivencia armónica
11. Sexualidad responsable y protegida
12. Adicciones y violencia
13. ¿Quiénes recibieron la capacitación? **(No leer las opciones, esperar que responda y marcar la o las opciones que se asemejen a su respuesta)**
14. Director
15. Miembros del Comité de establecimientos de consumo escolar
16. Miembros del Comité de activación física
17. Responsable del establecimiento de consumo escolar
18. Profesor
19. Profesor de educación física
20. Vendedores y/o expendedores de alimentos y bebidas
21. Alumnos
22. Madres/Padres o tutores de familia

**CARTILLA NACIONAL DE SALUD**

Finalmente, le haré dos preguntas relacionadas a la Cartilla Nacional de Salud (**No leer las opciones, esperar que responda y marcar la respuesta).**

1. ¿Le entregaron las cartillas nacionales de salud para sus alumnos?
2. Sí
3. No (Finaliza el cuestionario)
4. ¿Distribuyó las cartillas nacionales de salud a los profesores?
5. Sí
6. No

**¡Muchas gracias por su participación!**
